# mSWI/SNF (BAF) Complexes Are Indispensable for the Neurogenesis and Development of Embryonic Olfactory Epithelium

**DOI:** 10.1371/journal.pgen.1006274

**Published:** 2016-09-09

**Authors:** Christina Bachmann, Huong Nguyen, Joachim Rosenbusch, Linh Pham, Tamara Rabe, Megha Patwa, Godwin Sokpor, Rho H. Seong, Ruth Ashery-Padan, Ahmed Mansouri, Anastassia Stoykova, Jochen F. Staiger, Tran Tuoc

**Affiliations:** 1 University Medical Center, Georg-August-University, Goettingen, Germany; 2 Max-Planck-Institute for Biophysical Chemistry, Goettingen, Germany; 3 Department of Biological Sciences, Institute of Molecular Biology and Genetics, Seoul National University, Seoul, Korea; 4 Sackler Faculty of Medicine, Department of Human Molecular Genetics and Biochemistry, Tel Aviv University, Tel Aviv, Israel; 5 DFG Center for Nanoscale Microscopy & Molecular Physiology of the Brain (CNMPB), Goettingen, Germany; University of California San Diego, UNITED STATES

## Abstract

Neurogenesis is a key developmental event through which neurons are generated from neural stem/progenitor cells. Chromatin remodeling BAF (mSWI/SNF) complexes have been reported to play essential roles in the neurogenesis of the central nervous system. However, whether BAF complexes are required for neuron generation in the olfactory system is unknown. Here, we identified onscBAF and ornBAF complexes, which are specifically present in olfactory neural stem cells (oNSCs) and olfactory receptor neurons (ORNs), respectively. We demonstrated that BAF155 subunit is highly expressed in both oNSCs and ORNs, whereas high expression of BAF170 subunit is observed only in ORNs. We report that conditional deletion of BAF155, a core subunit in both onscBAF and ornBAF complexes, causes impaired proliferation of oNSCs as well as defective maturation and axonogenesis of ORNs in the developing olfactory epithelium (OE), while the high expression of BAF170 is important for maturation of ORNs. Interestingly, in the absence of BAF complexes in BAF155/BAF170 double-conditional knockout mice (dcKO), OE is not specified. Mechanistically, BAF complex is required for normal activation of Pax6-dependent transcriptional activity in stem cells/progenitors of the OE. Our findings unveil a novel mechanism mediated by the mSWI/SNF complex in OE neurogenesis and development.

## Author Summary

The stepwise differentiation of oNSCs to immature and mature ORNs is driven by a defined set of transcription factors. Yet, how these transcription factors act in concert with epigenetic and chromatin-remodeling cofactors to orchestrate OE neurogenesis is unknown. In this study, we identified subunits of chromatin-remodeling mSWI/SNF (BAF) complexes in oNSCs and ORNs. Our results indicate that early formation of the OE depends on the presence of BAF complexes. Our study highlights BAF155 and BAF170 as key factors that control specification, self-renewal of oNSCs and neuronal maturation in the developing OE. Furthermore, we uncover a novel mechanism in which the interaction between BAF155-containing BAF complexes with the transcription factor Pax6 determines OE neurogenesis.

## Introduction

Olfaction—the sense of smell—influences many primitive behaviors, including feeding, reproduction, social interactions, and fear responses [1,2,3]. The olfactory system is mainly composed of the olfactory epithelium (OE), the olfactory bulb (OB), and the olfactory cortex. The mouse OE arises from olfactory placodes (OPs) during early embryogenesis [1,2,3]. Whereas neurogenesis is initiated very early in development, the majority of olfactory epithelial cells are proliferating olfactory neural stem cells (oNSCs) until E11.5 (S1A and S1B Fig) [4,5]. Beyond E12.5, the OE begins to become organized into apical, middle and basal layers, with proliferative activity limited to the apical and basal layers (S1A and S1C Fig) [4]. The majority of apical cells are proliferative glial-like sustentacular (SUS) cells, whereas basal cells are precursors of olfactory receptor neurons (ORNs), also called olfactory sensory neurons (OSNs) [1,2,6,7]. ORNs project their axons directly to the OB (S1D Fig) [8,9]. Among non-neuronal–lineage cells of the OE, SUS cells are thought to arise from oNSCs, but details of this process are still under investigation (S1A and S1C Fig) [10].

Cell-fate specification involves numerous steps in which the directed expression of genes initiates a lineage-specific program. Key transcriptional regulators of embryonic and adult OE neurogenesis have been described [1,2,6,10,11,12]. The stepwise differentiation of oNSCs is driven by a defined set of transcription factors, including Pax6 (paired box 6), Sox2 (SRY-box 2), FoxG1 (forkhead box G1), Mash1 (mammalian achaete scute homolog-1), Ngn1 (neurogenin 1), NeuroD1 (neurogenic differentiation 1), and Lhx2 (LIM homeobox 2) [12,13,14]. Yet, how these transcription factors act in concert with epigenetic and chromatin-remodeling cofactors to orchestrate neuronal specification is unknown.

Various chromatin remodelers have been identified as key players in neurogenesis, including those classified as Brg1/Brm-associated factor (BAF) complexes [15,16]. In mouse brain, BAF complexes comprise about 15 subunits [17], including Brg1 or Brahma as ATPase core units [18], and BAF155 and BAF170 as scaffolding subunits [19,20]. Depending on the specific BAF subunit combination, BAF complexes are important for NSC maintenance, neuronal differentiation, and maturation of neurons in the central nervous system [17,19,21,22]. However, the composition of BAF complexes and the link between BAF complex-mediated chromatin changes and specific transcriptional programs that control neurogenesis in OE are not known.

In this study, we identified subunits of BAF complexes in oNSCs and ORNs and a switch of a conserved subunit during their differentiation program. Using a conditional deletion approach in the mouse in which BAF155 and BAF170 deficiency is restricted to Foxg1-positive cells, we examined the phenotypes of single mutants (BAF155cKO, BAF170cKO) and dcKO mutants for BAF155 and BAF170, subunits in both onscBAF and ornBAF complexes, during development of the OE. We found that BAF155 and BAF170 play essential roles in specification, oNSC proliferation and ORN maturation of developing olfactory epithelium. Furthermore, we identified a genetic interaction between the BAF155 subunit of the chromatin-remodeling BAF complex and the transcription factor Pax6, a key regulator in the development of the olfactory system. We found that mice with conditional deletion of *BAF155* (*BAF155*cKO) display a phenotype similar to that of Pax6-deficient mutants, including malformation of the OE and OB, as well as impaired proliferation of oNSCs and maturation of ORNs in the developing OE. The severity of *BAF155*cKO embryo phenotypes was dramatically enhanced by concurrent heterozygous loss of one Pax6 allele. Mechanistically, BAF complex is required for activation of Pax6-dependent transcriptional activity in stem cells/progenitors of the OE. Our findings suggest a Pax6-mSWI/SNF complex-mediated molecular mechanism in the ontogeny of the OE.

## Results

### Identification of BAF complexes in the developing OE

**Fig 1:**
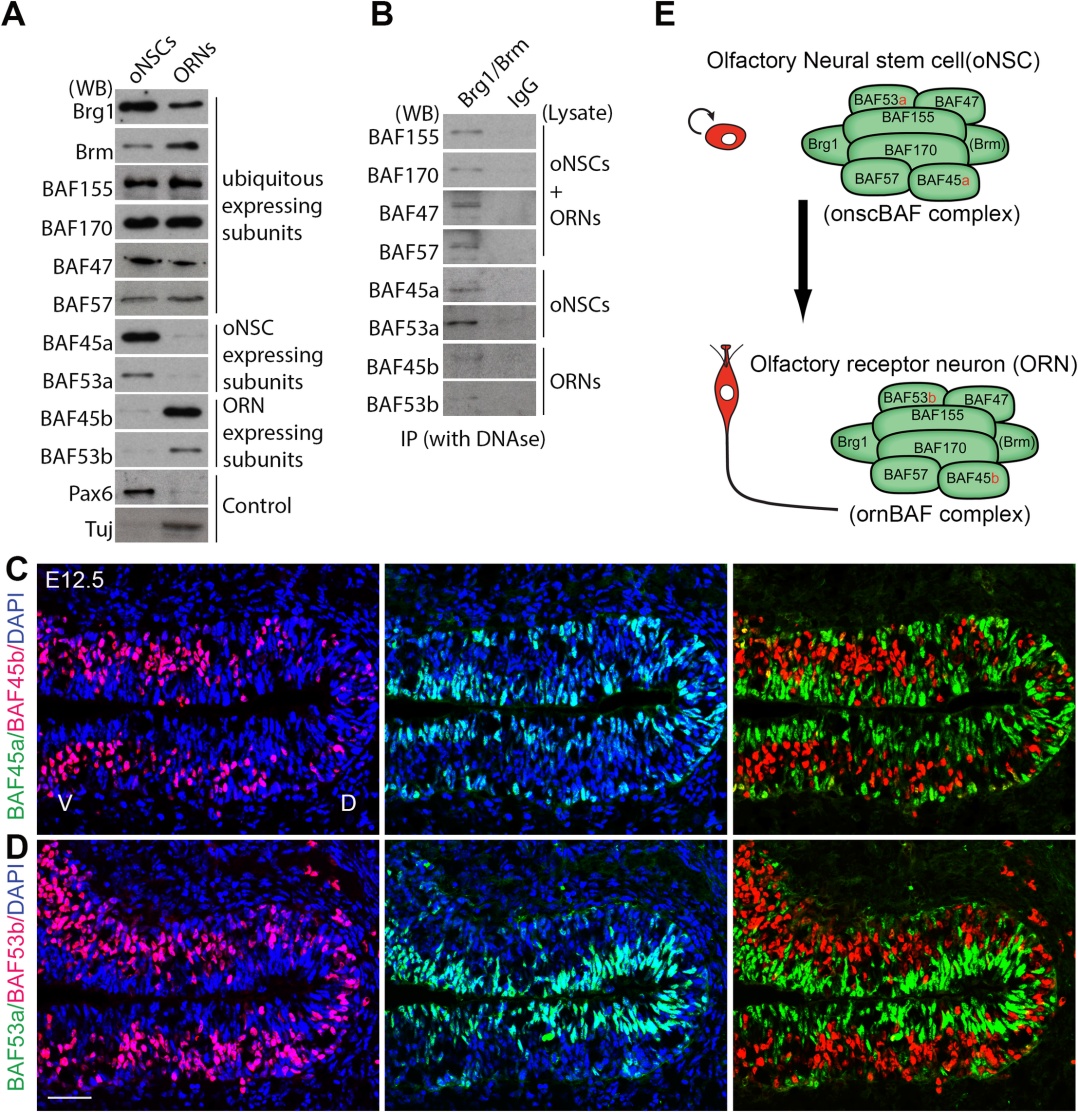
Subunit switching in the BAF complex during differentiation of oNSCs to ORNs. (A) Expression of BAF subunits in oNSCs and ORNs. Cell lysates were prepared from cultured oNSCs and ORNs, and blotted with BAF subunit-specific antibodies. (B) Co-immunoprecipitation of nuclear extracts from cultured oNSCs and ORNs using anti-Brg1/Brm antibodies and Western blot analyses with antibodies against BAF subunits revealed that BAF subunits integrate into Brg1/Brm-based BAF complexes. (C, D) Double-immunostaining of coronal sections of the OE from E12.5 mice revealed non-overlapping expression of oNSC-specific BAF45a/BAF53a subunits and ORN-specific BAF45b/BAF53b subunits in the developing OE. (E) A switch in subunits of BAF complexes during differentiation from oNSCs to ORNs. Abbreviations: oNSC, olfactory neural stem cell, ORN, olfactory receptor neuron; D/V, dorsal/ventral. Scale bars = 50 μm (C, D).

Using proteomics approach, a previous study identified BAF subunits in the newborn (P1) mouse brain [17]. In essence, the resulting data indicated a switch in the subunit composition of a neuronal progenitor (np) BAF complex to a neuron (n) BAF complex. In addition to ubiquitous subunits (Brg1, Brm, BAF170, BAF155, BAF57, BAF47), the npBAF complex contains the specific subunits, BAF45a and BAF53a. As neural progenitors differentiate into neurons, these subunits are replaced by the homologs, BAF45b, BAF45c and BAF53b, in the nBAF complex [17]. We examined whether such a subunit switch also takes place during differentiation from oNSCs to ORNs. To this end, we generated primary cultures of oNSCs from mouse embryos [23,24,25]. Similar to primary NSCs derived from the cortex [22,26,27], oNSCs cultivated in serum-free culture medium supplemented with EGF (epidermal growth factor) and FGF2 (fibroblast growth factor-2) divided rapidly [23], expressing the stemness markers Pax6 and Nestin (Fig 1A). When grown in Neurobasal culture medium supplemented with glutamate and B27, they differentiated into neurons [23], as evidenced by upregulated expression of the neuronal markers β-III tubulin (Tuj) and HuC/D [23] (Fig 1A).

To examine expression of BAF subunits in oNSCs and ORNs, we performed Western blot analyses on lysates from cultured cells using antibodies against BAF subunits. We found that BAF subunits present in oNSCs and ORNs (Fig 1A) were similar to those in NSCs and neurons in the brain [17], namely subunits with ubiquitous expression (Brg1, Brm, BAF170, BAF155, BAF57, BAF47), oNSC-specific subunits (BAF45a and BAF53a), and ORN-expressed subunits (BAF45b, and BAF53b). Our Brg1/Brm co-immunoprecipitation and Western blot analyses using BAF subunit-specific antibodies confirmed that these BAF subunits integrate into Brg1/Brm-containing complexes in oNSCs and ORNs (Fig 1B). An examination of the expression of the identified specific subunits in oNSCs and ORNs showed that BAF45a and BAF53a were present in proliferative Pax6^+^Sox2^+^ cells in both basal and apical sides of the developing OE, whereas BAF45b and BAF53b co-staining was observed in Ctip2^+^HuCD^+^ ORNs in the intermediate zone of the developing OE at E12.5. Importantly, expression of oNSC-specific BAF45a/BAF53a subunits and ORN-specific BAF45b/BAF53b subunits was found to be mutually exclusive (Fig 1C and 1D). Taken together, our data indicate that oNSCs and ORNs contain their own BAF complexes, hereafter termed onscBAF and ornBAF complexes, with their own specific subunits (Fig 1E).

### Dynamic expression of BAF155 and BAF170 in developing olfactory epithelium and in their deficient mutants

Previous findings revealed that the expression of BAF155 is high in embryonic stem cells (ESCs) and cortical progenitors in VZ, whereas its expression is generally down-regulated during differentiation [22,28,29]. In contrast, BAF170 is expressed at lower level in stem/progenitor cells and its higher expression is found in differentiated cells [22,28,29]. In addition, loss of BAF170 in cortex-specifc BAF170cKO mutants led to incorporation of addition BAF155 molecules into BAF complexes in cortical cells [22,30].

**Fig 2:**
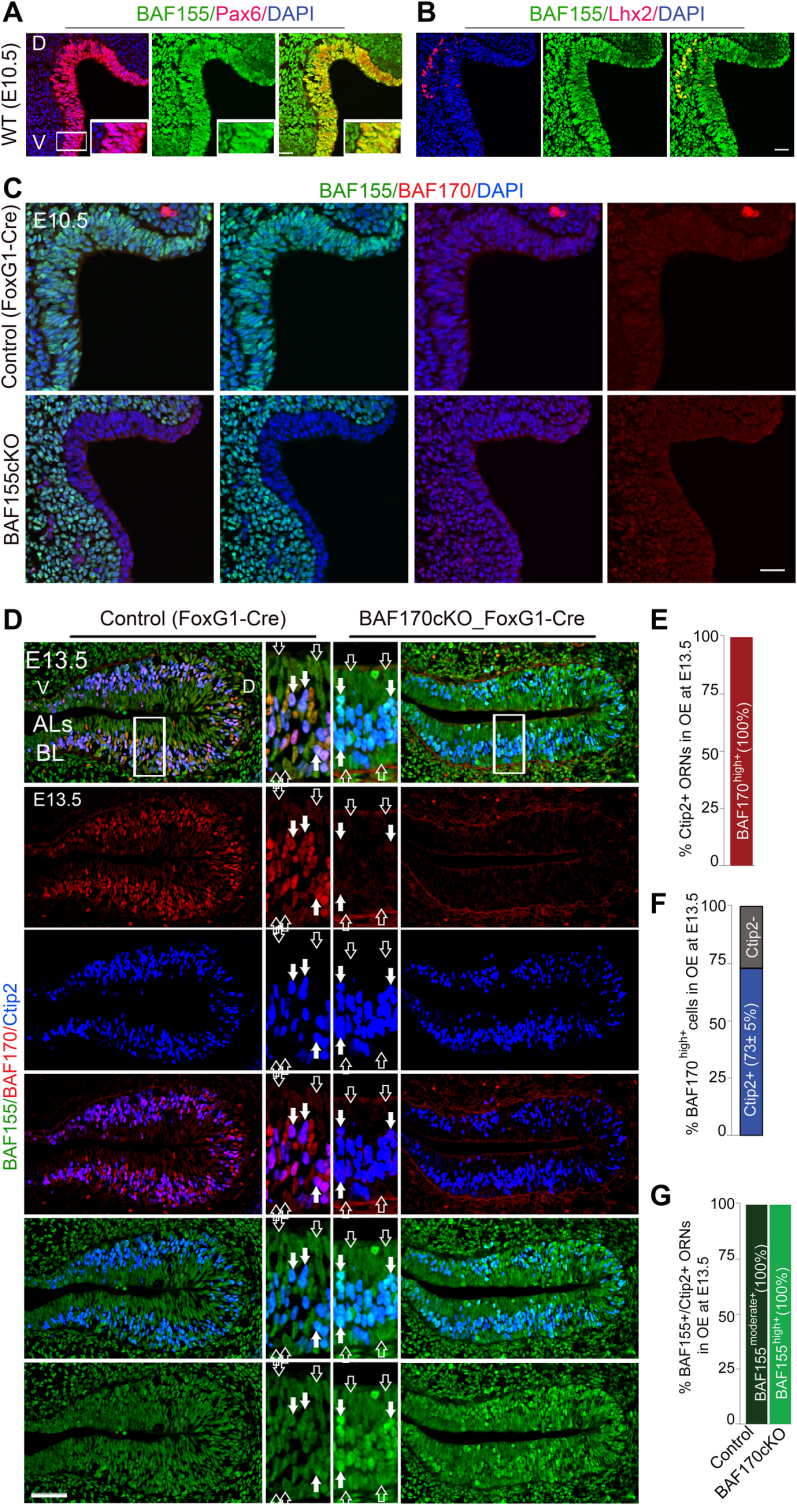
Expression of core BAF155 and BAF170 subunits in olfactory cell subtypes and in their mutants. (A–D) Representative images from coronal sections of the OE at the indicated embryonic stages (D, dorsal; V, ventral). (A, B) Double-IHC analysis of the ubiquitous BAF155 subunit showed wide expression in the developing OE, including in Pax6^+^ oNSCs (A) and Lhx2^+^ ORNs (B) (yellow in overlaid images; see also S2 Fig for additional analyses). (C-G) IHC (C, D) and statistical analyses (E-G) is used to compare expression of BAF155 and BAF170 in control, in *BAF155cKO_FoxG1-Cre* at E10.5 (C) and in *BAF170cKO_FoxG1-Cre* OE at E13.5 (D). A moderate expression of BAF155 was observed in all cells types at E10.5 and E13.5 (control OE in C, D, G, arrows, BAF155^moderate+^ cells). BAF170 is expressed at low level at E10.5 OE and at BL and ALs of E13.5 OE (Control OE in C-F, emtry arrows, BAF170^low+^ cells), whereas most of cells with high expression of BAF170 were Ctip2^+^ neurons (control OE in D-F, filled arrows, BAF170^high+^ cells). Notably, loss of BAF170 causes a upregulated expression of BAF155 in Ctip2^+^ neurons (D, G filled arrows, BAF155^high+^ cells), but not in Ctip2^-^ cells (D, G, emptry arrows). Abbreviations: D/V, dorsal/ventral; BL, basal layer; ALs, apical layers; ILs, intermediate layers. Scale bars = 25 μm (A, B, C) and 50 μm (D).

As a considerable difference to other cell lineages, our immunohistochemical analysis (IHC) revealed a high expression of BAF155 not only in dividing oNSCs (E10.5, and BL at E13.5), and SUSs (ALs at E13.5), but also in neurons (ILs at E13.5) in developing OE. We found that BAF155 is expressed widely, including Pax6^+^Sox2^+^ oNSCs, pHH3^+^ proliferating progenitors, Mash1^+^ neuronal progenitors, Tuj^+^HuCD^+^Ctip2^+^Lhx2^+^ ORNs, and Pax6^+^Sox2^+^ oNSCs at the basal layer and in proliferative glia-like SUS cells in apical layers from E12.5 onward (Figs 2A and 2B and S2). Similar to its low expression in mitotic ESCs [28] and cortical progenitors [22,30], a faint BAF170 expression was detected in OE at E10.5, in basal (BL) and apical layers (ALs), which contain mostly proliferative oNSCs and SUSs (Fig 2C and 2D, empty arrow). The expression of BAF170 become upregulated in intermediate layers (ILs), which contain Ctip2^+^Lhx2^+^HuCD^+^ neurons (Fig 2D–2F, filled arrows).

To examine expression of BAF155 and BAF170 in each mutant and their roles in development of olfactory epithelium *in vivo*, we crossed mice bearing floxed alleles of *BAF155* (*BAF155*^*fl/fl*^) [31] with mice expressing *FoxG1-*Cre [32], generating *BAF155*cKO_*FoxG1*-Cre mutants. In the *FoxG1*-Cre mouse line [32], Cre recombinase is driven in discrete structures of the head, including the telencephalon, eyes, and OE. Starting from E8.5, Cre activity was found in the anterior neural ridge, olfactory placodes, and OE [33]. Thus, the *FoxG1*-Cre line is appropriate for Cre recombinase activity in early development of OP/OE at which their differentiation from cephalic ectoderm can be detected [32,33,34]. The efficiency of Cre-mediated recombination was verified in OE sections at E10.5–E13.5 by IHC using anti-BAF155 and anti-BAF170 antibodies (Fig 2C and 2D). The results revealed a complete loss of BAF155 and BAF170 in the developing OE and telencephalon of *BAF155*cKO and *BAF170*cKO embryos, respectively, confirming *BAF155* and *BAF170* knockout (Fig 2C and 2D).

Using IHC, we next examined the expression of BAF155 and BAF170 in the respective single knockout mutant of the other BAF subunit, we performed IHC against BAF155 and BAF170 in tissue of BAF155cKO (Fig 2C), BAF170cKO embryonic OE (Fig 2D). We found a comparably-low expression of BAF170 between control and BAF155cKO_FoxG1-Cre OE at E10.5, implicating that BAF155 does not control the expression of BAF170 (Fig 2C). This is also consistent with our observation in the developing cortex.

Because of the easily-distinguishable specific low expression of BAF170 in BL (BAF170^low+^ oNSCs) as well as in ALs (BAF170^low+^ SUSs), and its high expression in ILs (BAF170^high+^ neurons), we studied whether the expression of BAF155 is affected in the BAF170cKO_FoxG1 OEs at E13.5. We did not observe any obvious difference in BAF155 expression between control and BAF170cKO mutant OE in Ctip2^negative^ oNSCs in BL and Ctip2^negative^ SUSs in ALs, where normally BAF170 expression was low (Fig 2D, empty arrows). Remarkably, loss of BAF170 led to an enhanced expression of BAF155 in Ctip2^+^ neurons in ILs, where normally BAF170 expression was high (Fig 2D and 2G, filled arrows). Thus our data indicated that BAF170 controls expression of BAF155, whereas the loss of BAF155 does not affect the expression level of BAF170 in developing olfactory epithelium.

### Dysgenesis of OE in *BAF155*cKO embryos

Given the high presence of the BAF155 subunit in both onscBAF and ornBAF complexes, we investigated the role of BAF155 in the development of OE during neurogenesis and neuronal maturation.

**Fig 3:**
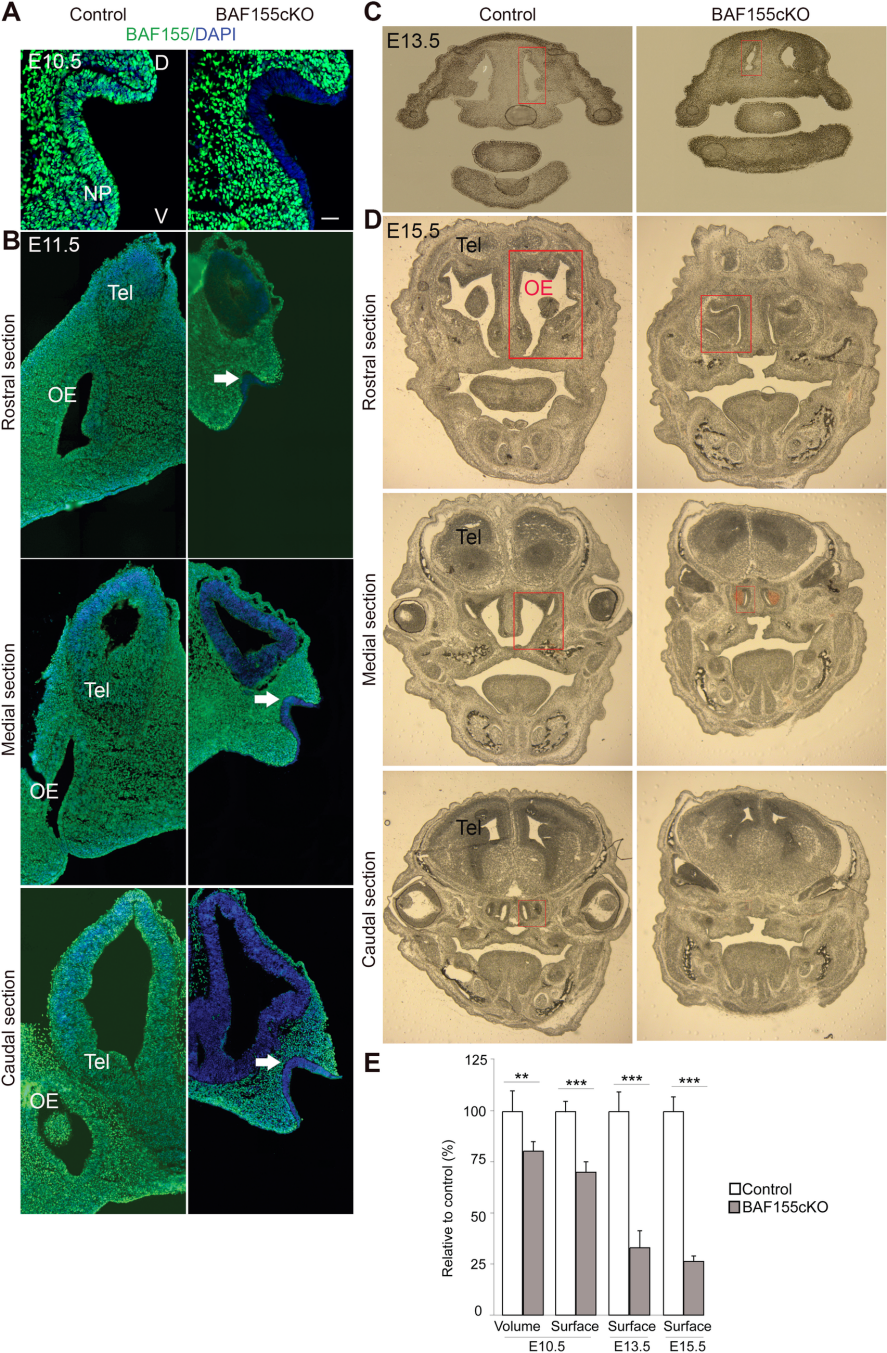
Histological analysis of the control and *BAF155*cKO developing OE. (A, B) Images of sections from the nasal pit (NP; arrow) at E10.5, and in the rostral, medial and caudal OE at E11.5 immunostained for BAF155. Note that, at E11.5, the NP has invaginated into the nasal cavity in controls, but has not yet been initiated in mutants. (C, D) Images show coronal cryosections of control and *BAF155*cKO embryos at E13.5 and E15.5. Note the smaller OE in mutants compared with controls. (E) Quantification of OE volume and surface area at E10.5 in A (also see S1 Movie), and surface area at E13.5 and E15.5, shown as red frames in C and D, respectively. Abbreviations: NP, nasal pit; OE, olfactory epithelium; Tel, telencephalon; D/V, dorsal/ventral. Values are reported as means ± SEM (*P < 0.05, **P < 0.01, ***P < 0.001). Scale bar = 25 μm.

The OE emerges from the nasal placode (NP) via a cascade of events that includes OP formation and cavity with fast proliferation and differentiation of oNSCs to configure epithelium layers. We histologically examined the OE of embryos (control and *BAF155*cKO) at different stages. Around E10.5–E11.5, the OP is formed and nasal cavities are established in control embryos (Fig 3A and 3B) [35]. At E10.5, mutants display a visibly smaller OP than controls in terms of both volume and surface area, as revealed by three-dimensional (3D) reconstruction analysis (Fig 3A and 3E and S1 Movie). In contrast, the olfactory pit and nasal cavities were not fully established in the mutant at E11.5 (Fig 3B). At E13.5 and E15.5, although the mutant OE was well formed, it was significantly smaller compared with control embryos (Fig 3C–3E). These findings indicate that BAF155 is necessary for normal formation of the OE early in development. The observed small-sized OE further suggests possible defects in progenitor proliferation or differentiation and/or increased apoptosis in *BAF155*cKO mutants.

### The BAF155 subunit is required for proliferation and maintenance of oNSCs

**Fig 4:**
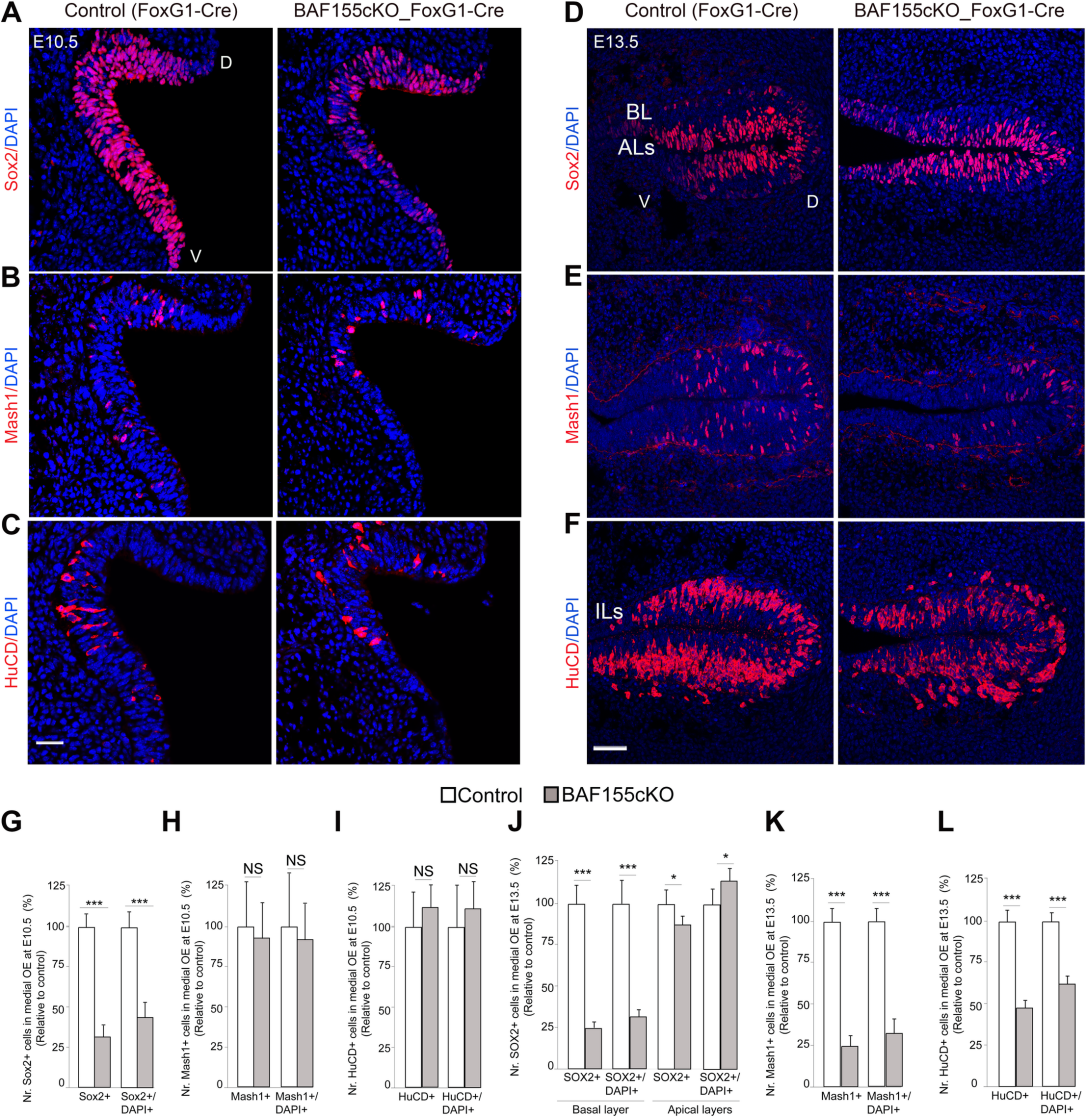
Depleted pool of oNSCs and diminished neurogenesis in embryonic *BAF155*cKO OE. (A–F) Images of OE sections from control and *BAF155*cKO embryos showing IHC detection of the oNSC marker Sox2 at E10.5 (A) and in the basal layer at E13.5 (D); the SUS cell marker in apical layers at E13.5 (D); the IP marker Mash1 at E10.5 (B) and E13.5 (E); and the neuronal marker HuCD at E10.5 (C) and E13.5 (F) (see S4 Fig for additional markers). (G–L) Statistical quantification of panels A–F is shown. Note the decrease in the number of Sox2^+^ oNSCs at both E10 and E13.5. Compared to controls, the number of Mash1^+^ IPs and HuCD^+^ ORNs was diminished at E13.5, but not E10.5, in BAF155-deficient OE. Remarkably, the loss of BAF155 did not affect the genesis of Sox2^+^ SUS cells at E13.5. Abbreviations: D/V, dorsal/ventral; BL, basal layer; ALs, apical layers; ILs, intermediate layers. Values are reported as means ± SEM (*P < 0.05, **P < 0.01, ***P < 0.001; NS, not significant). Scale bars = 25 μm (A–C) and 50 μm (D–F).

At early development stages (E10.5–E11.5), few OE cells from WT mice are neuronal progenitors (Mash1^+^) or immature neurons (HuCD^+^); instead, the majority are oNSCs (Sox2^+^Pax6^+^), which undergo rapid proliferation to expand their population (Fig 4A–4C and S1B Fig and S3A–S3L Fig). Early OE neurogenesis (E10.5) in *BAF155*cKO mice was apparently normal, based on the expression of the neuronal progenitor-specific markers Mash1 and Ngn1, and the immature ORN-specific markers HuCD, Tuj, and Lhx2 (Figs 4B, 4C, 4G and 4I and S3E–S3L). Strikingly, however, the *BAF155*cKO OE contained a significantly lower number of Sox2^+^Pax6^+^ oNSCs (Fig 4A and 4G and S3C and S3D Fig).

Under normal conditions, progenitors migrate to occupy the basal epithelium by E13.5 (S1C Fig). Moreover, the stem cell/progenitor cell population in the basal layer of the OE continues to produce new ORNs throughout life [1,2,6,36]. We next investigated the self-renewal of oNSCs and the generation of neuronal progenitors, ORNs, and SUS cells at E13.5. Similar to stage E10.5, we found that loss of BAF155 led to a severely diminished number of Sox2^+^Pax6^+^Nestin^+^ oNSCs within the OE basal layer (Fig 4D and 4J and S3M and S3N Fig). In contrast to E10.5, *BAF155*cKO OE at E13.5 contained a decreased number of neuronal progenitors (Mash1^+^Ngn1^+^) (Fig 4E and 4K), terminally—dividing neurogenic progenitors (NeuroD1^+^) (S3M and S3N Fig) and immature ORNs (HuCD^+^Tuj^+^GAP43^+^), which also exhibited reduced expression of the corresponding markers (Fig 4F and 4L and S3M and S3N Fig). Notably, the number of Sox2^+^Pax6^+^ cells in ALs, which are usually SUS cells, was not significantly different between mutant embryos and control littermates at E13.5 (Fig 4D and 4J). By IHC analysis, we examined the expression of additional SUS markers, including: Otx2 [7,37], Cytokeratin 18 (K18), REEP6 [38] (S4 Fig). Consistently, we found an equal number of Otx2^+^HuCD^-^ SUS cells and a similar expression of K18 and REEP6 as revealed by quantification of fluorescent signal between OE of control and BAF155cKO mutants at E13.5 and E15.5 (S4 Fig).

**Fig 5:**
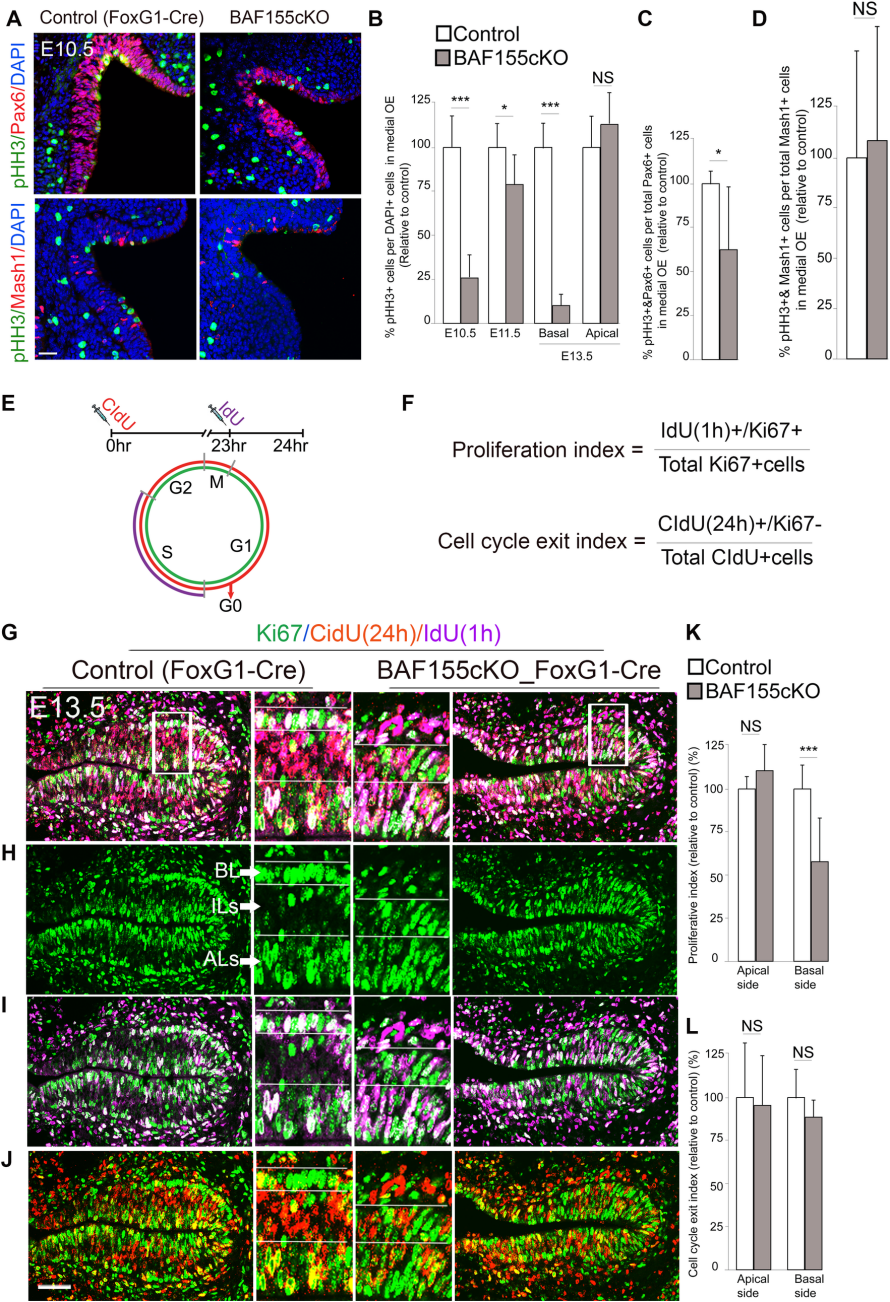
Cell cycle indexes of oNSCs in the developing OE show selective defects in *BAF155*cKO mutants. (A) Images of double-IHC for either the oNSC marker Pax6 or the IP marker Mash1 and the marker of mitotically active cells, pHH3, in E10.5 OE. (C, D) Statistical analyses showed that the loss of BAF155 led to a selective decrease in pHH3^+^ oNSCs at early stages (E10.5–E11.5) (B), Pax6^+^ oNSCs at 10.5 (C), and basal pHH3^+^ oNSCs at E13.5 (B), but not apical pHH3^+^ SUS cells (B) or pHH3^+^/Tbr2^+^ IPs (D). (E, F) Experimental paradigm in which mutant and control embryos were labeled with CIdU for 24 hours (to mark cells both in and exiting from the cell cycle) and IdU for 1 hour (to label progenitors in S-phase) by injection of the corresponding nucleoside analogs. (G–J) Images showing triple IHC at E13.5 for CIdU (red), IdU (magenta) and proliferation marker Ki67 (green) in control and *BAF155*cKO mutants. Middle panels: higher magnifications images of areas indicated by white boxes (G). (K, L) Statistical analyses of proliferation indexes (i.e., the number of IdU^+^/Ki67^+^ cells per total number of Ki67^+^ cells) for cells in panel I, showing a significantly lower value in mutant OE at the basal side, but not at the apical side (K). No significant differences were found in the exit index (i.e., number of CidU^+^/Ki67^-^ cells per total number of CidU^+^ cells) for cells in panel J between controls and mutants (L). Values are reported as means ± SEM (*P < 0.05, **P < 0.01, ***P < 0.001; NS, not significant). Scale bars = 25 μm (A) and 50 μm (G–J).

To ascertain if loss of functional BAF155 leads to alterations in cell proliferation, we immunostained for phosphorylated histone H3 (pHH3), a mitosis (M)-phase marker, and for integration of thymidine analogs, to detect cells in synthesis (S) phase and those exiting the cell cycle. At E10.5–E11.5, cells in M-phase were detected mainly at the apical surface of the OE in control animals. By contrast, very few pHH3^+^ cells were basally located (Fig 5A). To precisely quantify the number of pHH3^+^ cells at E10.5, we reconstructed the OE in serial sections (S1 Movie) and counted pHH3^+^ cells throughout the OE in mutant embryos and control littermates. This analysis showed that the loss of BAF155 severely diminished the number of pHH3^+^ cells in the OE at E10.5, and also reduced the number of these cells at E11.5, albeit to a lesser extent (Fig 5A and 5B). Furthermore, compared with controls, the ratio of pHH3^+^/Pax6^+^ M-phase oNSCs, but not pHH3^+^/Mash1^+^ M-phase intermediate progenitor cells (IPs, also named as transit amplifying progenitors), was lower in mutants at E10.5 (Fig 5A, 5C and 5D). Because oNSCs migrate to locate in the BL of OE from E13.5 onward, the pool of pHH3^+^ cells in the BL of *BAF155*cKO OE was severely depleted compared with that of controls at E13.5 (Fig 5B). Consistent with the preserved pool of dividing SUS cells, pHH3^+^ cells at the apical surface were not significantly altered between *BAF155*cKO and control OE at E13.5 (Fig 5B). Our findings imply that the loss of BAF155 selectively affects the proliferation of oNSCs, but not IPs or dividing SUS cells (S3 and S4 Figs).

To better characterize *BAF155* loss-of-function effects on proliferation and cell cycle exit of progenitors, we established quantitative proliferative and exit indexes in the developing OE using injection of thymidine analogs (IdU, CIdU) (Fig 5E and 5F), an experimental approach that has been widely used in the developing cortex [22,39,40]. Accordingly, cycling OE cells were pulse-labeled *in vivo* with CldU for 24 hours and with IdU for 1 hour. OE sections were triple immunostained at all stages of the cell cycle using antibodies for CIdU, to label both cycling progenitors and those that nascently exited from the cell cycle; IdU, to mark S-phase progenitors; and Ki67, a marker for proliferating progenitors. Sections of medial OE at E13.5 were chosen, because the basal layer (containing oNSCs), intermediate layers (comprising neurons), and apical layers (with SUS cells) of the medial OE are fairly distinguishable in controls at this time point (Fig 5G). As expected, in control OE, most Ki67^+^ proliferating cells and IdU^+^ cells in S-phase were found in the basal layer (oNSCs) and apical layers (SUS cells) (Fig 5H and 5I). A few CIdU^+^Ki76^+^ cells re-entering the cell cycle were also seen in the basal layer, and many such cells were detected in apical layers (Fig 5J, cells in yellow). In addition, the majority of CIdU^+^Ki76^-^ cells exiting the proliferative cycle and possibly becoming neurons were identified in intermediate layers (Fig 5J, cells in red). In contrast to control OE, the border between the basal layer and intermediate layers was not recognizable in mutant OE (Fig 5G–5J). We therefore determined cell cycle indexes at apical and basal sides, which include both the basal layer and intermediate layers. Statistical analyses revealed a significantly lower proliferative index in *BAF155*cKO OE at the basal side than in controls. However, the index was similar between mutant OE and control at the apical side. Although fewer neurons were seen in mutants compared with controls, surprisingly, cell cycle exit index values at both basal and apical sides were not significantly different between them. Finally, *BAF155*cKO OE displayed no apoptotic defects, as assessed by IHC detection of cleaved caspase 3 in OE tissue at E10.5 and E13.5 (S3K and S3L Fig). Thus, our data show that the BAF155 subunit is essential for proliferation of oNSCs. The decreased OE neurogenesis observed in *BAF155*cKO mutants at E13.5 appears to be the consequence of a depleted oNSC pool (S1A–S1C Fig).

### Control of neurogenesis in the OE by BAF155 involves activation of Pax6 transcriptional activity

**Fig 6:**
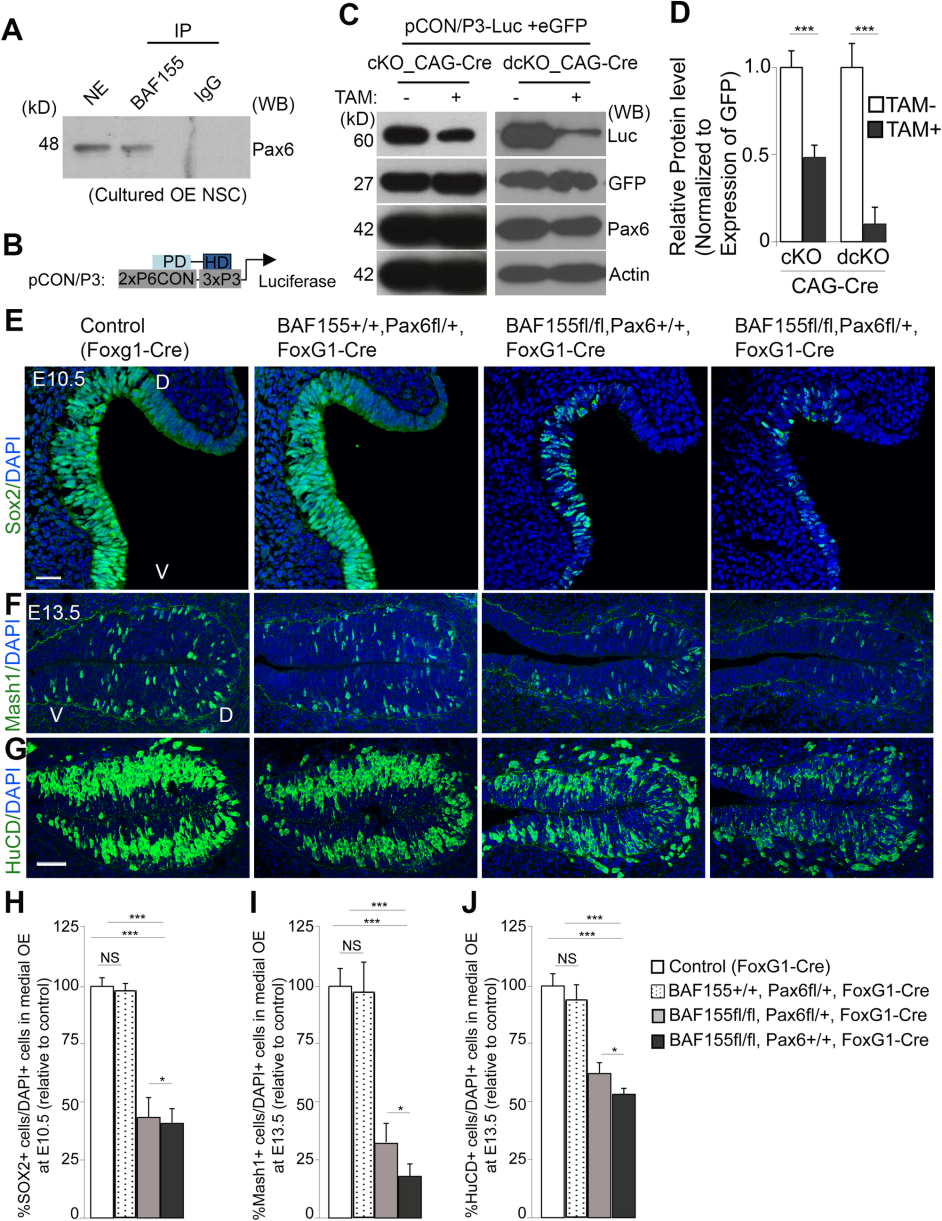
BAF155 and Pax6 synergistically control neurogenesis in embryonic OE. (A) Immunoprecipitation of BAF155 from cultured OE NSCs and Western blot analysis using an anti-Pax6 antibody demonstrated that BAF155 interacts with Pax6 in OE cells. (B–D) Cultured OE NSCs from control or *BAF155*cKO or *d*cKO embryos were electroporated with pCON/P3 (see B), a Pax6-dependent reporter construct, and an EGFP expression plasmid. After 2 days of *in vitro* culture (DIV), the electroporated cells were collected and analyzed by Western blotting with the indicated antibodies. Western blotting (C) and statistical analyses (D) indicated that compared to cultured OE cell control, the Pax6-dependent transcriptional activity was moderate (in *BAF155cKO* mutants) and severely diminished in dcKO mutants. (E–G) Images show IHC detection of the oNSC marker Sox2 (E), the neuronal progenitor marker Mash1 (F), and the post-mitotic neuron marker HuCD (G) in OE sections from the indicated transgenic embryos. (H–J) Statistical comparisons indicated increased decreased numbers of progenitors and neurons in the OE of *BAF155*cKO mice compared with control mice. These phenotypic features of *BAf155*cKO (*BAF155*^fl/fl^_*Pax6*^+/+^_FoxG1-Cre) mice were dramatically exacerbated by additional heterozygous loss of one *Pax6* allele (*BAF155*^fl/fl^_*Pax6*^fl/+^_*FoxG1*-Cre). Abbreviations: D/V, dorsal/ventral. Values are reported as means ± SEM (*P < 0.05, **P < 0.01, ***P < 0.001; NS, not significant). Scale bars = 25 μm (E) and 50 μm (F, J).

In previous studies, we and others found that Pax6 protein, a key player in OE development [41,42,43,44,45,46], interacts with multiple BAF subunits, including BAF170, BAF155 and Brm, in the cortex [22,47]. Here, we extended this investigation to examine whether BAF155 interacts with Pax6 in cultured oNSCs by performing co-immunoprecipitation assays using a BAF155 antibody and cultured OE cells. We confirmed that BAF155 indeed interacted with Pax6 in OE cells (Fig 6A). In addition, all Pax6^+^ cells in the developing OE were also immunoreactive to the BAF155 antibody (Fig 1C and S4A Fig). These data imply an interaction between Pax6 and BAF155 *in vivo*.

We then set out to determine whether the interaction between BAF155 and Pax6 affects Pax6-dependent transcriptional activity by using a generated Pax6-dependent reporter plasmid (pCON/P3) (Fig 6B) [26,48]. Mice harboring floxed alleles of *BAF155* [31] were crossed with tamoxifen-inducible *CAG*-CreER mice, in which CreER expression is driven by the ubiquitous CAG promoter [49], generating *BA155*cKO_*CAG*-CreER mutants. Primary oNSCs, subsequently isolated from *BA155*cKO_*CAG*-CreER embryos and cultured *in vitro*, exhibited efficient deletion of BAF155 two days after addition of 4-hydroxytamoxifen. *BA155*cKO_*CAG*-CreER oNSCs were nucleofected with plasmids expressing pCON/P3 and EGFP, and the expression level of luciferase was examined by Western blot analysis after 3 days of tamoxifen treatment. We found that loss of BAF155 (+tamoxifen) in *BAF155*cKO oNSCs led to reduced levels of luciferase expression compared with those from control cells (-tamoxifen) (Fig 6B–6D). Thus, our data indicate that BAF155 is required for activation of Pax6-dependent transcriptional activity.

To compare more directly the OE phenotype of BAF155 and Pax6 mutants during development *in vivo*, we generated *Pax6*cKO_*FoxG1*-Cre mice. The phenotype of *Pax6*cKO mutants was similar to that of homozygous *Small eye* mutants [50] and *Pax6*-null mice [51], characterized by the absence of eyes and OB, a smaller cortex, and altered dorso-ventral patterning (S5 and S6 Figs). Intrigued by the identified interaction between BAF155 and Pax6, we first examined whether BAF155 influences the morphology of the above mentioned cranial structures (S6 Fig). Strikingly, we found that *BAF155*cKO mice exhibited a phenotype similar to that of *Pax6*cKO mice, including the lack of eyes and OB, and reduced cortical size (S6A Fig). We probed for the expression of Reelin and NP-1, which mark the OB structure (S6B Fig, arrows), the OB is formed at E18.5 in both WT and *BAF155*cKO embryos, but outgrowth of the OB at the rostral-most part of the forebrain was only observed in WT mice, and not in *BAF155*cKO mice (S6B Fig, arrows).

To investigate whether the *BAF155* loss-of-function phenotype (diminished number of Sox2^+^ oNSCs, Mash1^+^ neuronal progenitors and HuCD^+^ neurons) became more severe with the additional loss of Pax6 function, we generated double *BAF155*cKO*/Pax6*cKO_*FoxG1*-Cre mutants (S5 Fig). Because Pax6-null mutants complete lack OE structures (S5B Fig), as previously reported [41,42,43,44,45,46], we investigated the phenotype of double-mutants homozygous for BAF155 and heterozygous for Pax6 (*BAF155*^*f*l/fl^_*Pax6*^fl/+^_*FoxG1*-Cre) and compared it with that of *BAF155*cKO mice (BAF155^*fl/fl*^_*Pax6*^+/+^_*FoxG1*-Cre) and controls (FoxG1-Cre). A similar approach has been used to investigate genetic interactions between the BAF complex and Sox10 in Schwann cell differentiation [52]. The pool of Sox2^+^ oNSCs at E10.5 and neuronal differentiation at E13.5 in OE in double-mutants were examined (Fig 6F–6K). A quantitative analyses revealed that the defects in the maintenance of Sox2^+^ oNSCs and genesis of Mash1^+^ IPs and HuCD^+^ neurons of the developing OE in *BAF155*cKO mutants were made more severe by the additional loss of one *Pax6* allele, which on its own had no effect on the number of oNSCs or ORNs in the OE [53] (Fig 6E–6J). Thus, both biochemical data and loss-of-function studies support the conclusion that the transcription factor Pax6 functionally interacts with the BAF complex via the BAF155 subunit to exert its regulatory effects on the proliferation of oNSCs and OE neurogenesis.

### Loss of mature ORNs and olfactory axon projections in *BAF155KO* mutants

**Fig 7:**
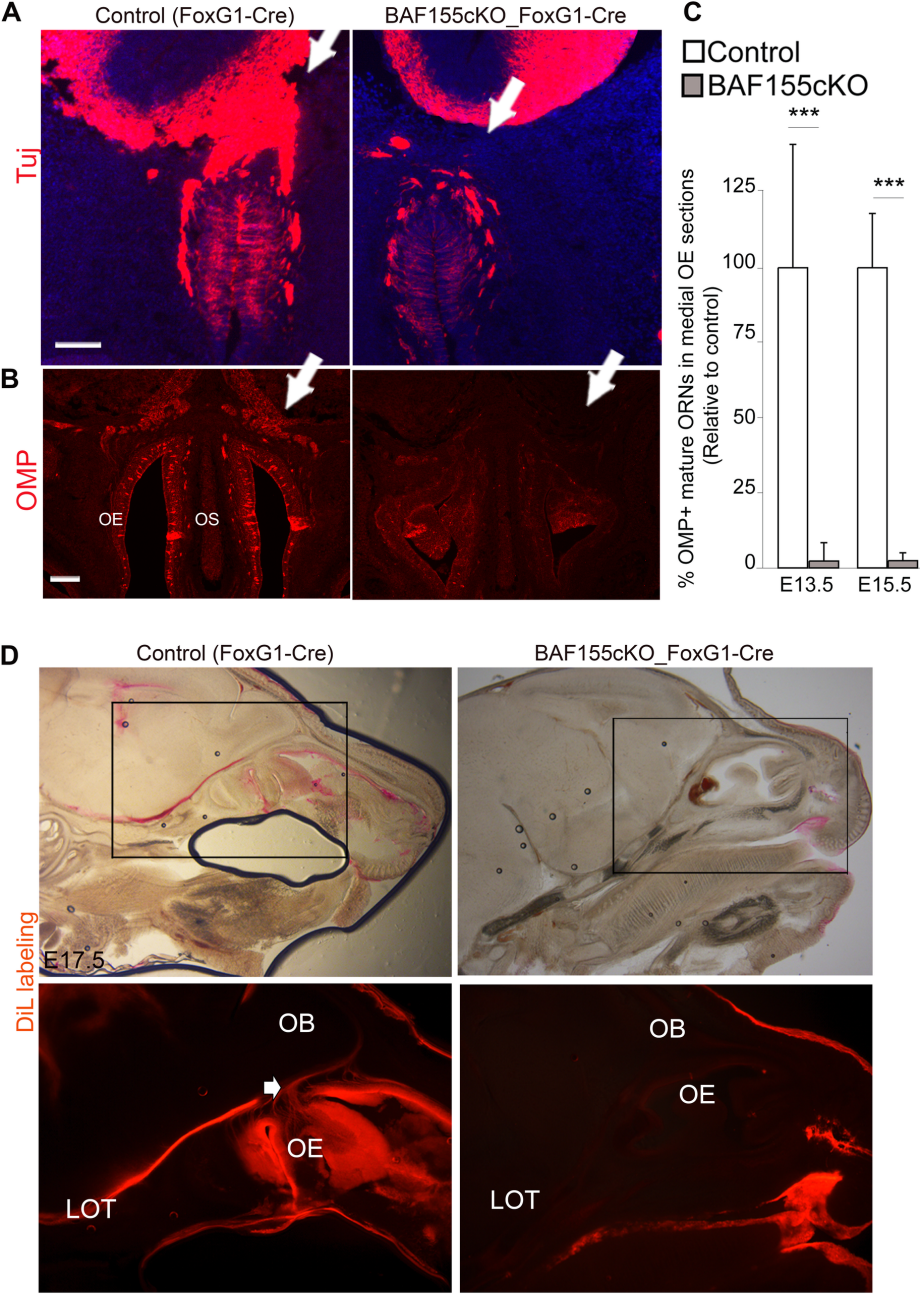
Absence of mature OSNs and their axonal tracts in BAF155-deficient mutants. (A) Coronal sections of the OE and frontal telencephalon of E13.5 and E15.5 embryos (S5C Fig) control and BAF155-deficient embryos were analyzed by IHC using anti-Tuj and anti-NCAM antibodies (S5C Fig). The axons of immature OSNs (Tul^+^NCAM^+^) reached the OB in control embryos, but failed to grow out and generate a fibro-cellular mass in BAF155-deficient embryos. (B) IHC analyses of cross sections through the head at E15.5 using an anti-OMP antibody, which labels mature OSNs and their axons, revealed a severely diminished number of OMP^+^ mature OSNs in the OE and a lack of axonal connectivity between the OE and rostral cortex in *BAF155*cKO mice compared with control mice (indicated by arrows). (C) Statistical analysis of OMP^+^ mature ORNs in control and *BAF155*cKO OE at E13.5 and E15.5. (D) Tracing the olfactory nerve with DiI at E17.5. Bright-field and fluorescent images are shown in upper and lower panels, respectively. In controls, the olfactory nerve and axons projecting to the OB are clearly outlined by DiI. In addition, these OB neurons, which have taken up DiI and project their exons via the LOT to the primary olfactory cortex, were also seen in control embryos (indicated by arrows), whereas no DiI-positive axonal projection pattern from the OE to the OB toward the olfactory cortex was detectable in the mutants. Abbreviations: ON, olfactory nerve, LOT, lateral olfactory tract. Values are reported as means ± SEM (***P < 0.001). Scale bars = 100 μm (A) and 150 μm (B).

We found that BAF155 is not required for early OE neurogenesis, as the numbers of Lhx2^+^Tuj^+^HuCD^+^ early ORNs were similar between *BAF155*cKO OE and controls at E10.5 (Fig 4 and S1B and S3 Figs). Notably however, expression of Ctip2, a key factor for the maturation of ORNs [54], was lower in BAF155 mutant OE compared with that in controls at all examined stages (E10.5–E13.5) (S7A and S7B Fig). Likewise, we found a lack of mature OMP^+^ ORNs at E13.5 and E15.5 compared with control OE (Fig 7B and 7C). These findings indicate that, although early neurogenesis is normal in the *BAF155*cKO OE, the BAF155 deficiency is nevertheless associated with a severe disturbance in the differentiation of mature ORNs (S1A and S1C Fig).

Upon differentiation, early-born ORNs in the developing OE project pioneer axons to the OB to direct axonal projections from the OE to the telencephalon [8,9,45,55,56]. In controls, the fascicle of ORN axons and cellular masses were positive for Tuj1 and N-CAM immunostaining [57,58,59,60] (Fig 7A and S7C–S7F Fig). As previously shown [57], ORNs advanced their N-CAM- and Tuj-stained axons via the cribriform plate into the forebrain (Fig 7A and S7C–S7F Fig, denoted by arrows). In contrast, no Tuj1^+^NCAM^+^ axonal tracts entering the forebrain and fewer Tuj1^+^NCAM^+^ cellular aggregates were identified in BAF155 mutants (Fig 7A and S7C–S7F Fig). Additionally, axonal projections from the OE to the OB were detected by OMP immunostaining in wild-type mice; however, no OMP staining was detected in *BAF155*cKO mutants (Fig 7B, arrows). These data suggest that BAF155 is required for the axogenesis of ORNs that project to the forebrain.

Neurons in the OE extend their axons to target glomeruli within the principal OB. Then, ORN axons connect with dendrites of OB neurons. Subsequently, axonal connections between these mitral cells in OB and the primary olfactory cortex form the lateral olfactory tract (LOT). Hence, mitral cells receive inputs from ORNs and relay them to the olfactory cortex through the LOT [8,9,61,62]. To determine if anterior telencephalic cells of mutants receive sensory inputs from ORNs, we injected a DiI crystal into the OE in the posterior part of the nasal cavity of embryos (control and mutant) at E17.5 and examined sagittal sections by fluorescence microscopy. A large DiI-labeled bundle of ORN axons entering the OB was detected in control embryos (Fig 7D). These DiI-positive axons of OB neurons also allowed visualization of the LOT. In contrast, these projections were not seen in mutant embryos (Fig 7D). These results indicate that BAF155 mutants have defects in maturation of ORNs, which fail to form normal axonal connections with the forebrain.

### High expression of BAF170 is required for appropriate maturation of ORNs

**Fig 8:**
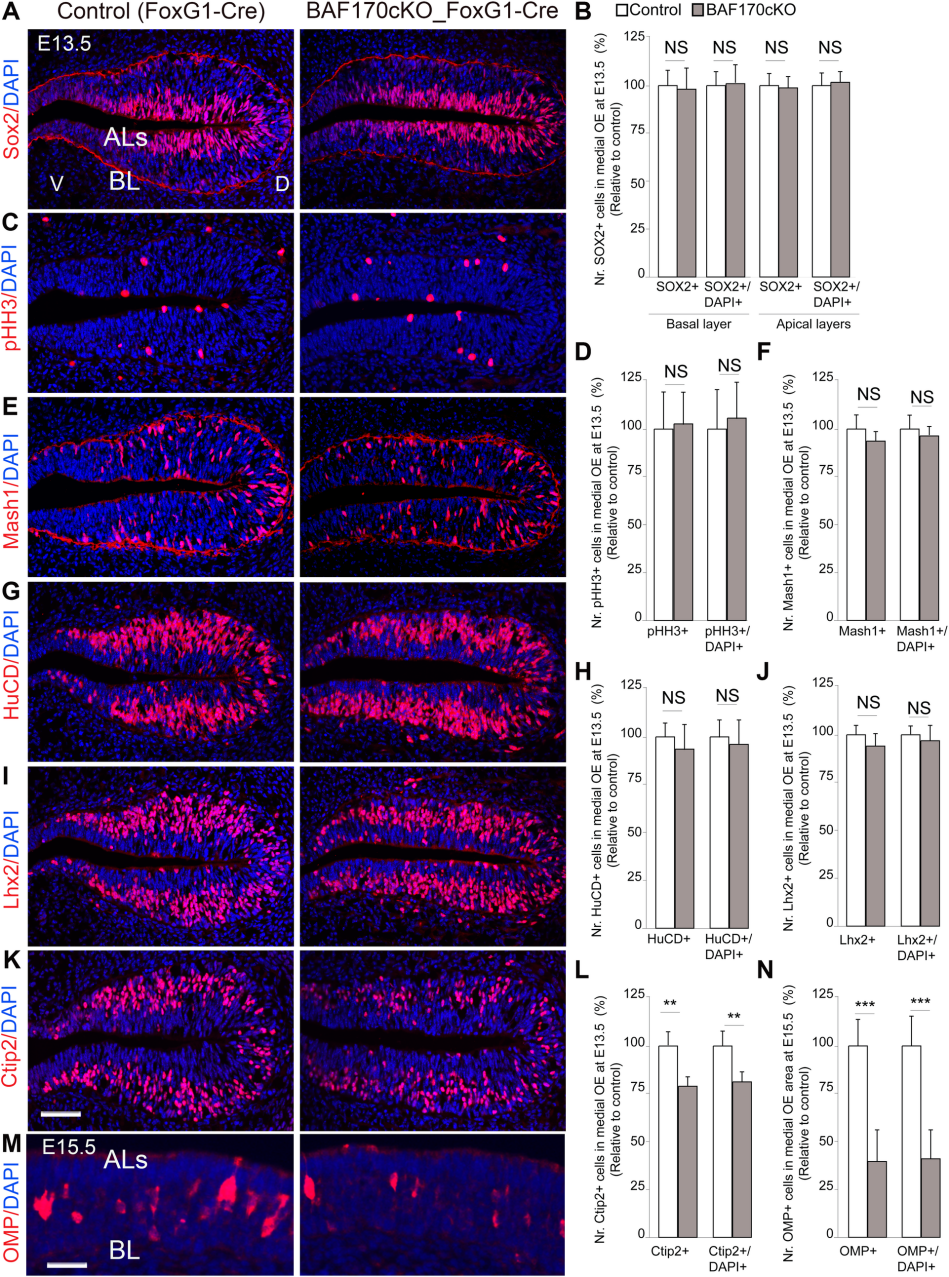
High expression of BAF170 is important for maturation of ORNs. (A, C, E, G, I, K, M) Images of IHC for markers: Sox2 (oNSCs, A), pHH3 (M-phase marker, C), Mash1 (IPs, E), HuCD, Lhx2 (immature ORNs, G and I respectively), Ctip2 (maturing ORNs, K) and OMP (mature ORNs, M) with OE sections from control and *BAF170*cKO embryos at E13.5 (A, C, E, G, I, K) and E15.5 (M). (B, D, F, H, J, L, N) Statistical quantification of panels (A, C, E, G, I, K, M) is shown. Compared to control, loss of BAF170 in *BAF170cKO* OE led to diminished number of Ctip2^+^OMP^+^ mature ORNs, but not pHH3^+^Sox2^+^ proliferating cells or Mash1^+^ IPs or HuCD^+^Lhx2^+^ immature ORNs. Abbreviations: D/V, dorsal/ventral; BL, basal layer; ALs, apical layers; ILs, intermediate layers. Values are reported as means ± SEM (*P < 0.05, **P < 0.01, ***P < 0.001; NS, not significant). Scale bar = 50 μm.

**Fig 9:**
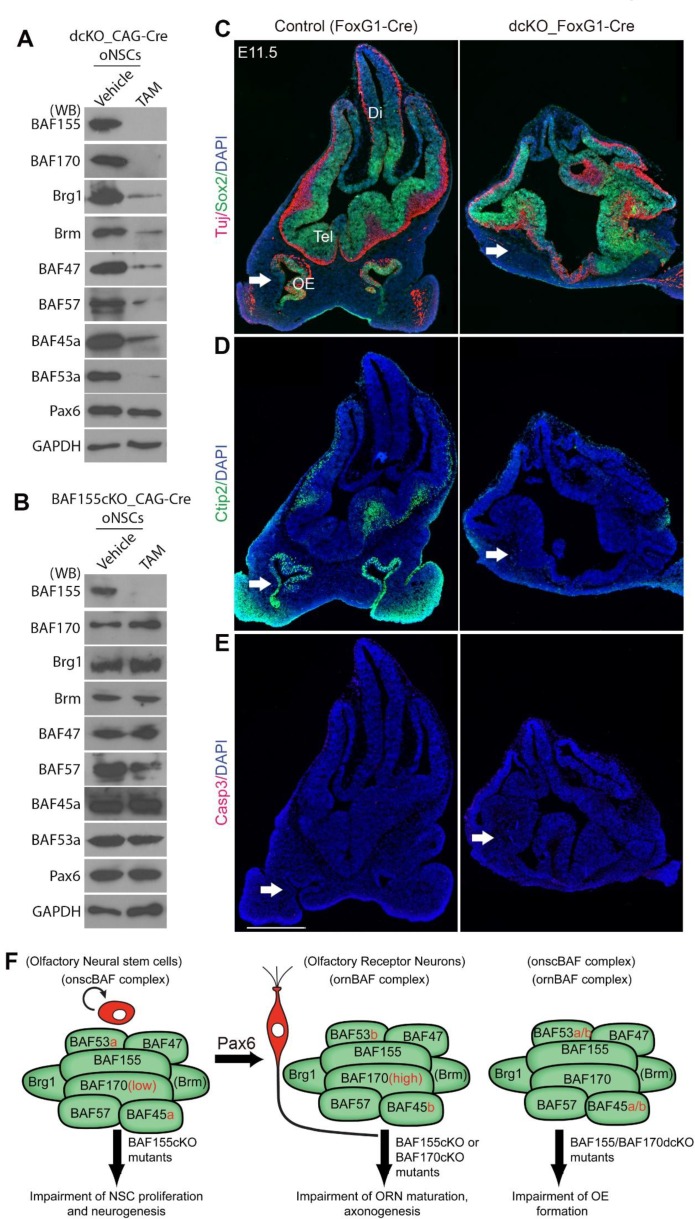
BAF155 and BAF170 expression are indispensable for OE development. (A, B) Expression of BAF subunits in *dcKO* (A) and *BAF155cKO* (B) cultured oNSCs was shown. (A) WB analyses revealed after 3 days of TAM treatment, expression of all known onscBAF subunits is nearly-completely lost in dcKO_*CAG*-Cre cultured oNSCs (A), whereas expression of most onscBAF subunits (except BAF57) is preserved in BAF155-deficient oNSCs (B). (C, D) IHC analyses using antibodies that specifically label oNSCs, SUS cells (Sox2 in C), immature ORNs (Tuj in C), and maturing ORNs (Ctip2 in D). *BAF155/BAF170*dcKO*_FoxG1*-Cre mutants lack OE at E11.5 (white arrows). (E) IHC analyses revealed no detectable Casp3^+^ apoptotic cells in both dcKO and control OE. (F) Schema shows the BAF subunits of BAF complexes identified in oNSCs and ORNs, as well as a summary of BAF mutant phenotypes in OE development. Abbreviations: OE, olfactory epithelium; Tel, telencephalon; Di, diencephalon. Scale bar = 500 μm.

Given the identified low-expression level of BAF170 in cells in BL and ALs and that of its upregulated expression in ORNs in developing OE, we sought to investigate the functional relevance of the dynamic of BAF170 expression in differentiation of oNSCs at E13.5 and E15.5. We first examined the pool of Sox2^+^pHH3^+^ mitotic oNSCs and SUSs at BL and ALs, respectively (Fig 8A–8D). In accordance with the low expression of BAF170 in these cell types, we found that the loss of BAF170 in cKO mutants did not affect the pools of Sox2^+^pHH3^+^ oNSCs and SUSs (Fig 8A–8D). Previously, we published findings that BAF170 controls genesis of intermediate progenitors (IPs) in developing cortex [22,30] and in postnatal dentate gyrus [63]. IHC analysis with antibodies for Mash1 (specific marker for IPs in OE), however, indicated no significant difference of Mash1^+^ IPs between control and mutant OE (Fig 8E and 8F). Likewise, as compared to the control, the number of HuCD^+^Lhx2^+^ immature ORNs is not altered in mutant OE (Fig 8G–8J) that BAF170 with its low expression level in mitotic cells, does not play a major role in proliferation and differentiation of OE progenitors. By examining expression of Ctip2, we found significally-diminished numbers of Ctip2^+^ maturing ORNs in BAF170cKO OE as compared to control at E13.5 (Fig 8K and 8L). Consistently, less number of OMP^+^ mature ORNs was identified in mutant OE than in the control (Fig 8M and 8N) at E15.5. Similar to *BAF155cKO* OE (S3K and S3L Fig) and *dcKO* mutants (see Fig 9), we did not find a significantly greater number of Casp3^+^ apoptotic cells in *BAF170cKO* OE as compared to control. Hence, our data indicated that during the switch from onscBAF complex to ornBAF complex, the proper maturation of ORNs requires the upregulated expression of BAF170.

### BAF complexes are indispensable for OE formation

Recently, we reported that conditional deletion of BAF155/BAF170 subunits in cortex-specific dcKO mice abolishes other BAF subunits in cortical tissue [19,20]. To examine whether BAF155 and BAF170 act as scaffolding subunits to maintain stability of onscBAF complex in OE cells, we crossed mice bearing floxed alleles of *BAF155* [31] and *BAF170* [22] (*BAF155*^*fl/fl*^_*BAF170*^*fl/fl*^) with mice expressing *FoxG1-*Cre [32], generating *BAF155*/*BAF170*dcKO_*FoxG1*-Cre mutants. Primary oNSCs subsequently were generated from BAF155/BAF170dcKO embryos. After 3 days of treatment with tamoxifen or vehicle as controls, cultured cells were collected for WB analysis to examine the expression level of BAF subunits (Fig 9A). Similar to cortical cells [19] and mouse embryos [20], we found that compared to control, the loss of all examined subunits of onscBAF complex was observed in tamoxifen-treated dcKO oNSCs (Fig 9A). Notably, loss of BAF155 alone did not affect stability of BAF complexes in OE cells as also seen in cortical cells [19], indicating that the presence of both BAF155 and BAF170 subunits were required for stability of BAF complexes (Fig 9B). In addition, we examined expression of Pax6 and Pax6-dependent reporter in cultured oNSCs without BAF155 or without both BAF155 and BAF170. The loss of either BAF155 or both BAF155 and BAF170 did not influence expression level of Pax6 (Fig 9A and 9B). However, as compared to control, Pax6-dependent transcriptional activity was diminished in *BAF155cKO* oNSCs and an even more profound effect was seen in dcKO oNSCs (Fig 6B–6D).

To characterize the phenotype of *BAF155*/*BAF170*dcKO embryos during early OE development (E9.5, E10.5 and E11.5), we first performed IHC analysis using an antibody against Sox2 (primordial oNSC marker) (Fig 9C, S8A and S8B Fig). Strikingly, Sox2^+^ cells were found only in telencephalon (Tel), but not in olfactory placode (OP) or epithelium (OE) of dcKO mutants at all examined stages (Fig 9C, S8A and S8B Fig, arrows). We reasoned that the absence of Sox2^+^ cells in mutant OE was possibly due to premature neuronal differentiation, which led to a depleted pool of Sox2^+^ oNSCs. The results from our performed IHC study with antibodies against Tuj, HuCD (immature neuronal marker), and Ctip2 (maturing neuronal marker), however, also revealed a complete lack of HuCD^+^Tuj^+^Ctip2^+^ neurons in dcKO olfactory placode/epithelium (Fig 9D and 9E, S8C Fig, arrows). Furthermore, similar to the dcKO phenotype in early cortical development [19,20], we failed to detect a significant increase in Casp3^+^ apoptotic cells in the telencephalon or OE of dcKO mutants compared with controls (Fig 9C). These findings indicated that in the absence of BAF complexes in BAF155/BAF170dcKO embryos, olfactory placode/epithelium is not specified.

In summary, our data indicate that high expression of BAF155 in oNSCs, and high level of BAF155 and BAF170 in ORNs is required for proper proliferation of oNSCs and maturation of ORNs (Fig 9F). In addition, low expression of BAF170 in ESCs, in developing cortex, and in OE progenitors together with presence of BAF155 are required to maintain the stability of all distinct BAF complexes in ESCs, cortex [19,20] and in oNSCs (this study).

## Discussion

We identified SWI/SNF (BAF) complexes in oNSCs (onscBAF complex) and ORNs (ornBAF complex), and investigated their roles in the development of the OE. We showed that expression of two scaffolding subunits, BAF155 and BAF170, is critical for oNSC proliferation, neuronal maturation and formation of the OE. We further demonstrated that control of neurogenesis in the OE by BAF complexes involves activation of Pax6 transcriptional activity.

### Chromatin remodeling SWI/SNF (BAF) complexes control development of the olfactory system

The roles of epigenetic and chromatin factors are beginning to be understood in brain development, whereas these key regulators have only recently been investigated in OE development [19,64,65,66,67]. Having previously established the essential roles of BAF complexes in the development of the central nervous system [15,17,19,22,30,68,69], we here determined the composition of BAF complexes in oNSCs and ORNs and investigated functions of such BAF complexes in the development and neurogenesis of embryonic OE.

Our examination of the expression pattern of core BAF subunits (Brm, Brg1, BAF47, BAF155, BAF170 and BAF250) in the developing OE revealed that these subunits are widely expressed, including in oNSCs, SUS cells, neuronal progenitors, and ORNs. These findings suggest that BAF complexes control a wide range of events in OE development. Recently, we presented evidence that elimination of both core subunits, BAF155 and BAF170, causes proteasome-mediated degradation of the entire BAF complex [19,20]. In addition, dcKO_*FoxG1*-Cre embryos, in which Cre is active during very early OE development and throughout telencephalic development (E8.5), exhibited a complete loss of the telencephalic structure at E16.5 [19,20]. We further found that OP/OE is not specified (Fig 9, S8 Fig), implicating that BAF complexes are crucial for OE development.

The differentiation of oNSCs to ORNs requires both an intrinsic program and extrinsic cues. Studies using knockout mice and cell-lineage tracing methods have indicated that this process comprises various intermediary stages, which are outlined by using molecular markers. Sox2^+^Pax6^+^ oNSCs produce committed IPs (Mash1^+^) [4,5,13,38], which in turn become neuronal precursors (Ngn1^+^ NeuroD1^+^). Neuronal precursors are capable of differentiating into immature neurons (Tuj^+^HuCD^+^), which will eventually transform into OMP^+^ mature ORNs. In addition to identifying ubiquitous subunits, we also found that a subunit switch that occurs during the differentiation of cycling progenitors to neurons appears to be conserved between the brain and the OE [17] (Fig 1). The onscBAF complex in oNSCs, and possibly proliferating glia-like SUS cells, contains BAF45a, BAF53a and BAF170^low^. As oNSCs exit the cell cycle, these subunits are replaced with homologs, BAF45b, BAF53b and upregulated expression of BAF170 (BAF170^high^) in the ornBAF complex in ORNs is found (Fig 1G). The NSC-specific subunits, BAF45a and BAF53a, are required for the renewal properties of multiple progenitor cells [17], whereas the neuron-expressing subunits, BAF45b and BAF53b, are essential for neuronal maturation in the brain [21]. Future investigations of the roles these BAF subunits in the development of OE are warranted.

Given the constant expression of BAF155 and upregulated expression of BAF170 in ORN differentiation, we characterized the phenotype of *BAF155*cKO and *BAF170*cKO mutants. We show here that deletion of BAF155 resulted in progressive depletion of the oNSC population and a subsequent reduction in the number of neurons at later stages. The role of BAF155 and the onscBAF complex in regulating the self-renewal and proliferative activity of NSCs is also reflected by the reduced number of Sox2^+^Pax6^+^ oNSCs as well as the decreased proliferation index in the BAF155-deficient developing OE. In contrast to oNSCs, the loss of BAF155 did not affect the population of Mash1^+^Ngn1^+^ neuronal progenitors or Tuj^+^Lhx2^+^HuCD^+^ pioneer neurons at E10.5, or the exit index. These data indicate that BAF155 is not required for early neurogenesis and differentiation of oNSCs to ORNs. Interestingly, BAF155 does not appear to be a general regulator of cellular proliferation in the developing OE as glia-like SUS cells proliferated normally in the absence of BAF155. As a result, the loss of BAF155 did not cause a change in the number of Sox2^+^Pax6^+^ SUS cells in ALs. Therefore, BAF155 is a key neurogenic factor that regulates the proliferation of oNSCs, but not that of SUS cells. Whereas NSCs of the OE mainly generate ORNs, considerable evidence suggests that they also produce the glial-like SUS cells in ALs [7,70,71,72,73,74]. Nevertheless, a full understanding of the possible differentiation of oNSCs into SUS cells will require further investigation [10]. It will also be interesting to investigate the role of BAF155 in cell-fate determination using fate-mapping approaches with an oNSC-specific Cre line.

Our analysis of neuronal maturation and the trajectories of ORN axons in *BAF155*cKO mutants showed that mutants have a thinner OE owing to a reduced number of immature ORNs. Remarkably, these ORNs failed to differentiate into mature OMP^+^ ORNs. In addition, we found that ORN axons, which express Tuj, N-CAM and OMP and take up DiI, were largely lost in the mutants. Differentiating ORNs develop axonal processes and eventually participate in neural circuits. Their axons enter the anterior telencephalon and connect with dendrites of mitral cells in glomeruli of the mouse OB [75,76]. Whether development of OB depends on the normal axonal projections of OSNs is still under debate [44,56,77,78], the lack of connections between OSN axons and mitral cells might have contributed to the lack of OB formation in BAF155 mutants (S1D Fig).

The characterization of *BAF170cKO* phenotype indicated that the high expression of BAF170 plays important role in maturation of ORNs. Nevertheless, the effect of depletion of BAF170 on neuronal maturation of ORN seems to be milder as more Ctip2^+^OMP^+^ mature ORNs were found in *BAF170cKO* OE than that in BAF155cKO OE. A possible explanation might be that the upregulated expression of BAF155 subunit in *BAF170cKO* OE might partially compensate for the loss of BAF170 during ORN maturation.

### Interplay between chromatin-remodeling BAF complexes and the transcription factor Pax6 in OE neurogenesis

Distinct BAF complexes are known to bind to and cooperate with tissue-specific transcription factors to modulate gene expression in different cell types, reflecting the widespread role of BAF complexes in the development of numerous organs [15,79]. As we come to identify the roles of different transcription factors in OE development, we also need to understand how the activity of these transcription factors is regulated. In this context, we present molecular and genetic evidence that the BAF155 subunit of the chromatin-remodeling BAF complex serves an essential role in olfactory neurogenesis by potentiating Pax6-dependent transcriptional activity.

In early development, expression of Pax6 is found in the frontonasal domain of the head, including the OP, from which the OE arises [41,80,81,82]. Consequently, olfactory placode development is massively compromised in Pax6-null mutants of mouse and rat [44,50,80,81,82,83]. In Pax6 mutants, the OE as well as ORNs and their axonal projections are totally absent [44,80,81,83]. These lines of evidence support the critical role of Pax6 in orchestrating distinct developmental events in the genesis of the olfactory system.

In this investigation, using a conditional inactivation system for BAF155 and Pax6, we found that *BAF155* and *Pax6* mutants have similar phenotypic features, including dysgenesis of olfactory structures. Compared with control embryos at E10.5, fewer Pax6^+^ cells were found, possibly reflecting a diminished pool of oNSCs in *BAF155*cKO developing OE rather than indicating direct control of Pax6 expression by BAF155. In support of this interpretation, IHC analyses of cranial sections of E15.5 *BAF155*cKO mice showed no obvious changes in the expression of Pax6 in the cortex or SUS cells (S4A Fig) [22]. In addition, loss of BAF155 or both BAF155 and BAF170 in cultured *BAF155cKO_CAG-CreER* or *dcKO_CAG-CreER* oNSCs does not affect the expression level of Pax6 (Fig 6C and 6D, Fig 9A and 9B). In previous studies, we and others found that, during neurogenesis of the embryonic and adult brain, Pax6 recruits the SWI/SNF complex via interactions with multiple BAF subunits, namely Brg1, Brm, BAF155 and BAF170, to control the expression of Pax6 target genes [22,30,47]. In addition, our reporter assay revealed that BAF155 is required for activation of Pax6-dependent transcription in stem cells/progenitors of the olfactory neuroepithelium (Fig 6B–6D), as we observed also in developing cortex. These findings suggest that, unlike the interaction between BAF170 and Pax6 in cortical development [22], BAF155 may positively control the expression of Pax6-regulated genes. In addition, we found that not only do *BAF155*cKO and *Pax6*cKO mice have a similar defective phenotype, the phenotype of *BAF155*cKO mice was also exacerbated by the additional loss of one *Pax6* allele. Taken together, our findings imply a link between BAF155-dependent chromatin changes and the Pax6-dependent transcriptional program that controls development of the olfactory system.

In summary, we have identified the composition of chromatin-remodeling mSWI/SNF (BAF) complexes in oNSCs and ORNs. Our findings demonstrated that BAF complexes play essential role in specification, self-renewal of oNSCs and neuronal maturation in the developing OE. Furthermore, we uncover a novel mechanism in which the link between BAF complexes and transcription factor Pax6 determines OE neurogenesis. Therefore, the functional interaction between BAF complexes and Pax6 disclosed here may provide insight into how oNSCs acquire ORN and SUS fates, and thus will contribute to establishing protocols for differentiatiion of these cell lineages from pluripotent oNSCs.

## Materials and Methods

### Ethics statement

Animals were handled in accordance with guidelines of the German Animal Protection Law and with the permission of the Niedersächsisches Landesamt für Verbraucherschutz und Lebensmittelsicherheit (LAVES) (approval number: AZ/14/1636).

### Animal care and transgenic mice

Floxed Pax6 [84], floxed BAF155 [31], floxed BAF170 [22] *FoxG1*-Cre [32], and *CAG*-Cre mice [85] were maintained in a C57BL6/J background. Animals were treated in the guidance of the local animal protection law.

### Plasmids and antibodies

Descriptions of plasmids and antibodies used are detailed in S1 Text.

### Protein-protein interaction assay

*In vivo* immunoprecipitation with anti-BAF155 antibodies was performed as previously reported [19,22].

### IHC and western blotting

IHC and Western blotting were performed as previously described [26,39].

### Culture and neuronal differentiation of oNSCs

oNSCs were prepared from the OE of E17.5 and E18.5 mice, as described previously [23,24]. A detailed description is presented in S1 Text. For reporter assays, cultured OE cells were electroporated with plasmids using a Mouse NSC Nucleofector Kit and a nucleotransfection device (Amaxa). Luciferase assays were carried out as described previously [26,86].

### DiI labeling

Five control and five homozygous mutant mouse embryos (E17.5) were euthanized by cervical dislocation and directly transferred into 1x phosphate-buffered saline (PBS). Embryo heads were cut off and transferred to 4% paraformaldehyde (PFA). A 3-mg/ml solution of DiI (1,1’-dihexadecyl-3,3,3’-tetramethylindocarbocyanine perchlorate) in methanol was injected into the nasal cavities of each embryo under a microscope using a 30-gauge needle. A total of 50 μl DiI solution was applied to each cavity. After injection of DiI solution, the nasal cavities were sealed with 2% low-melting point agarose containing 2% PFA. The tissue was then transferred to 2% PFA in 1% low-melting point agarose and kept for 8 weeks in the dark at 4°C. Finally, embryo heads were cut into serial 150-μm sections in a sagittal plane. Pictures were taken using an Olympus SZX12 microscope and processed using CellF Imaging and Adobe Photoshop CS3.

### 3D reconstruction and cell counting

3D images of the OE were constructed using Neurolucida software version 11.03. Consecutive sections (25 μm each) of E10.5 wild-type and *BAF55*cKO OEs were imaged in rostro-caudal order. Contours were drawn in each section based on the expression of OE-specific markers. The 3D reconstruction was produced from whole-stack contours. The contours were placed into sets for left and right OEs. The volume analysis was done using Neurolucida Explorer v. 11.03.

### Imaging, quantification, and statistical analyses

Imaging was captured using an Axio Imager M2 (Zeiss) with a Neurolucida system, and confocal fluorescence microscopes (TCS SP5; Leica). Images were further processed with Adobe Photoshop. WB and IHC signal intensities were quantified by using ImageJ software, as described previously [19,26]. Statistical comparisons were carried out using Student’s *t*-test. The results are presented as means ± SEM. A detailed description of quantitative analysis methods is presented in S1 Text. All details of statistical analyses for histological experiments are presented in S1 Table.

## Supporting Information

S1 Fig Descriptive schemes for the development of wild-type *BAF155*cKO OE and OB. (A) After neuronal cell-fate determination, oNSCs differentiate into immature ORNs, then mature ORNs. Whether non-neuronal SUS cells are also generated from oNSCs in the developing OE is under investigation. These cell types can be distinguished by several markers, as indicated. Loss of BAF155 specifically affects the proliferation of oNSCs and the differentiation of immature ORNs to mature ORNs, resulting in a depleted pool of oNSCs and a loss of mature ORNs in the *BAF155*cKO OE (see also B and C). (B) At early stages (E10.5–E11.5), many oNSC, a few IPs, and some immature ORNs are found in the OE. Because the loss of BAF155 does not influence OE neurogenesis, the number of IPs and immature ORNs is equal between control and *BAF155*cKO OE in these early-stage embryos. (C) At later stages (E12.5–E15.5), the OE is structured into apical (with SUS cells), middle (comprising IPs and ORNs), and basal (containing oNSCs) layers. The ablation of BAF155 does not affect the proliferation or total number of SUS cells (see also A). The decrease in OE neurogenesis observed in late-stage (E13.5–E15.5) *BAF155*cKO mutant embryos is apparently a consequence of loss of the oNSC pool. (D) A schematic comparing the morphology of the OE, OB, and axonal projections from the OE to the OB via the LOT toward the primary olfactory cortex between control and *BAF155*cKO mutant embryos.
(TIF)

S2 Fig Expression of BAF155 in early developing mouse OE. BAF155-expressing cells were characterized in coronal sections of the OE at E10.5 (A) and E13.5 (B) by double-label immunofluorescence microscopy using an antibody for BAF155 (green) in combination with antibodies against the following marker proteins (red): Sox2, in oNSCs at E10.5 (A), and in oNSCs in the basal layer and glia-like SUS cells in apical layers at E13.5 (B); pHH3 in progenitor cells at M-phase of the cell cycle; Mash1 in neuronal progenitors; and Tuj, Ctip2, and HuCD in post-mitotic neurons. Abbreviations: BL, basal layer; ALs, apical layers; D/V, dorsal/ventral. Scale bars = 25 μm (A) and 50 μm (B).
(TIF)

S3 Fig Phenotype of *BAF155*cKO mutants in early developing OE. (A–L) IHC (A, C, E, G, I, K) and quantitative (B, D, F, H, J, L) analyses indicate that the loss of BAF155 leads to a diminished number of Pax6^+^ oNSCs and pHH3^+^ cells in M-phase of the cell cycle, without affecting Ngn1^+^ committed neuronal progenitors, Tuj^*+*^Lhx2^*+*^ neurons, or Casp3^+^ apoptotic cells at E10.5. Note that quantification of pHH3^+^ cells (B) was done in the entire OE using 3D reconstruction (see also S1 Movie). (M, N) IHC (M) and quantitative (N) analyses revealed that the expression of Nestin, NeuroD1, Tuj, and GAP-43 at E13.5 is lower in BAF155-deficient OE than in controls. Values are reported as means ± SEM (*P < 0.05, **P < 0.01, ***P < 0.001; NS, not significant). Scale bars = 50 μm (A–K) and 50 μm (M).
(TIF)

S4 Fig Expression of SUS markers in developing OE of *BAF155*cKO mutants. (A–E) Images of IHC with OE section from E13.5 (A-C), E15.5 (D-E) from control and BAF155cKO_FoxG1-Cre embryos and antibodies against: HuCD to labeling ORNs (A), Otx2 to stain both nucleus and cytoplasm of SUS cells [7,37,87] (A), K18 as well as REEP6 to mark SUS cells on the apical layer as well as their basal projections [38]. (F) Quantitative analyses of panels (A-E) indicated that the loss of BAF155 leads to a diminished number of HuCD^+^ ORNs (A, see also Fig 4), whereas expression of SUS markers: (Otx2, K18, REEP6) were preserved in BAF155-ablated OE as compared to controls. Values are reported as means ± SEM (*P < 0.05, **P < 0.01, ***P < 0.001; NS, not significant). Scale bars = 50 μm (A–C) and 150 μm (D).
(TIF)

S5 Fig Generation of transgenic lines for *BAF155*cKO and *Pax6*cKO knockout in the eye, OE, and cortex. (A) Double-IHC analysis of cross sections of E15.5 heads using anti-BAF155 (green) and anti-Pax6 (red) antibodies indicated a loss of BAF155 expression in the entire forebrain, OE, and eyes of *BAF155*cKO mice. Although cortex, eye and OE structures are abnormally formed in *BAF155*cKO mice, the expression of Pax6 is largely preserved. (B) Double-IHC analysis with anti-Pax6 (red) and anti-Mash1 (green) antibodies on cross sections of E13.5 heads revealed a loss of Pax6 expression in the entire head structures of *Pax6*cKO mice. Similar to Sey/Sey mice, OE (marked by expression of Mash1, arrow) is lost in *Pax6*cKO mice.
(TIF)

S6 Fig Abnormal morphology of the OB in BAF155-deficient mutants. (A) Images of brains (dorsal view) from control *and BAF155*cKO and *Pax6*cKO mice at E18.5. Unlike controls, *BAF155*cKO and *Pax6*cKO mice lack the OB (indicated by arrow in control). (B) Double-IHC analyses of E18.5 sagittal sections of the brain using anti-NP1 and anti-Reelin antibodies showed that OB formation is induced in the rostral-most telencephalon (arrows) in both control and mutants, but the outgrowth process of the OB is disturbed in *BAF155*cKO mice.
(TIF)

S7 Fig OE neurogenesis and axonogenesis in *BAF155*cKO mutants at E15.5. (A, B) IHC (A) and quantitative (B) analyses revealed the loss of Ctip2^+^ maturing ORNs during OE development (E10.5, E11.5, E13.5). (C–F) IHC (C, E) and quantitative (D, F for selected white boxes in C and E) analyses for the neuronal markers, Tuj (C, D) and N-CAM (E, F) showed reduced expression of the neuronal markers Tuj and N-CAM in the OE of BAF155 mutants at E15.5, and thinner bundles of axons compared with controls (indicated by arrows in C, E). In contrast to the case in controls, ORN axons in mutants do not make their normal contact with the forebrain, as evidenced by the gap between axonal bundles and the forebrain. Values are reported as means ± SEM (***P < 0.001). Scale bars = 50 μm (A) and 150 μm (C–E).
(TIF)

S8 Fig BAF155 and BAF170 expression are required for specification of olfactory placode. (A-C) Representative images show IHC analyses with coronal sections of olfactory placode (OP) from control and dcKO_FoxG1-Cre embryos at E9.5 (A) and E10.5 (B, C) with antibodies that specifically label primordial oNSC marker Sox2 (A, B) and immature ORN marker HuCD (C). IHC analyses revealed no detectable Sox2^+^ HuCD^+^ OP cells (pointed by arrows), implicating that OP/OE was not specified in dcKO embryos. Abbreviations: OP, olfactory placode; OE, olfactory epithelium; Tel, telencephalon; D/V, dorsal/ventral.
(TIF)

S1 Table Statistical analyses. (XLS)

S1 Movie 3D reconstruction of the E10.5 OE. (RAR)

S1 Text Supplemental experimental procedures. (DOC)
