# Supplementary figures and images for: mSWI/SNF (BAF) Complexes Are Indispensable for the Neurogenesis and Development of Embryonic Olfactory Epithelium
Source: PLoS Genet. 2016 Sep 9;12(9):e1006274. doi: 10.1371/journal.pgen.1006274 (PMC5017785; doi:10.1371/journal.pgen.1006274)

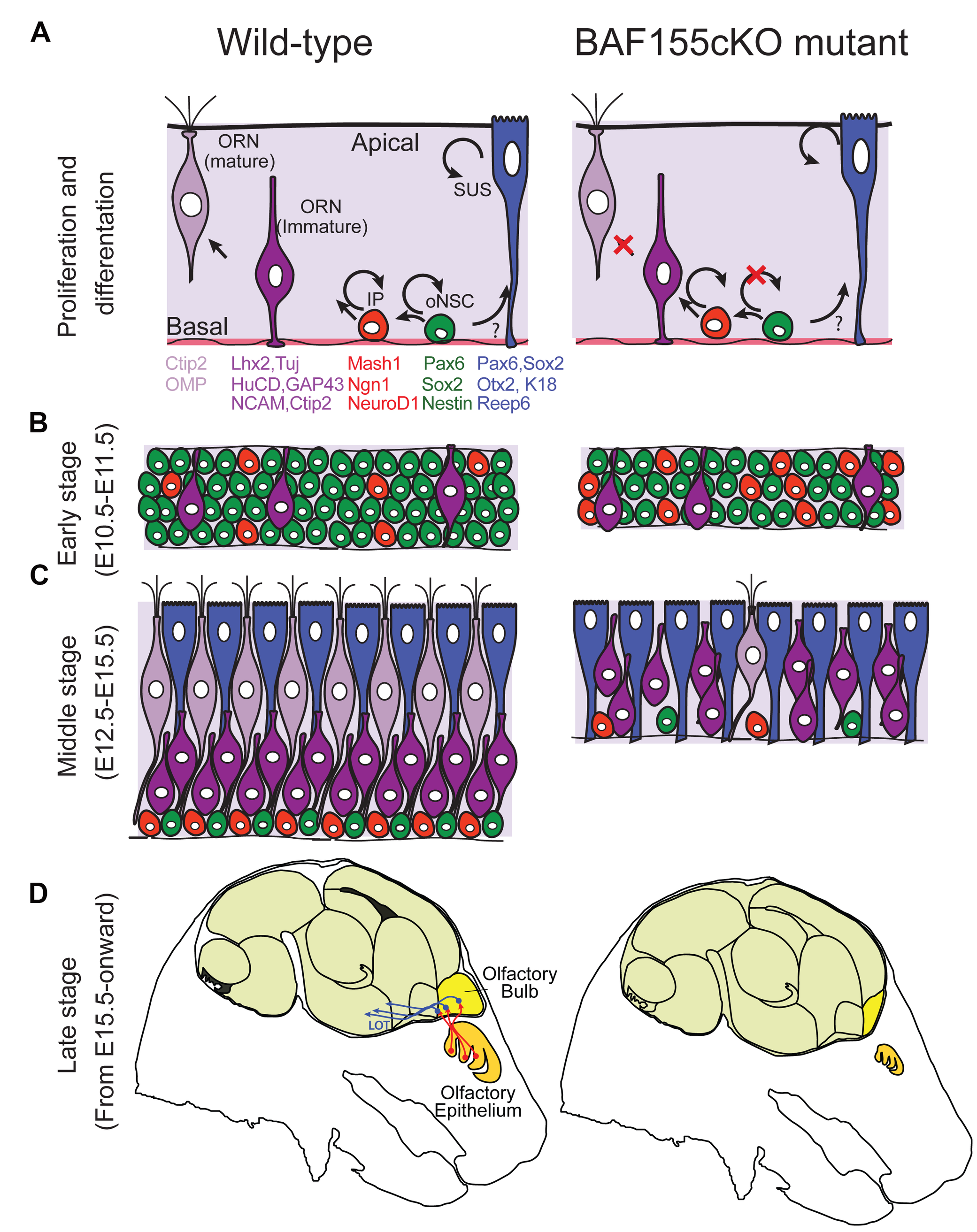

Supplement: S1 Fig — (A) After neuronal cell-fate determination, oNSCs differentiate into immature ORNs, then mature ORNs. Whether non-neuronal SUS cells are also generated from oNSCs in the developing OE is under investigation. These cell types can be distinguished by several markers, as indicated. Loss of BAF155 specifically affects the proliferation of oNSCs and the differentiation of immature ORNs to mature ORNs, resulting in a depleted pool of oNSCs and a loss of mature ORNs in the BAF155cKO OE (see also B and C). (B) At early stages (E10.5–E11.5), many oNSC, a few IPs, and some immature ORNs are found in the OE. Because the loss of BAF155 does not influence OE neurogenesis, the number of IPs and immature ORNs is equal between control and BAF155cKO OE in these early-stage embryos. (C) At later stages (E12.5–E15.5), the OE is structured into apical (with SUS cells), middle (comprising IPs and ORNs), and basal (containing oNSCs) layers. The ablation of BAF155 does not affect the proliferation or total number of SUS cells (see also A). The decrease in OE neurogenesis observed in late-stage (E13.5–E15.5) BAF155cKO mutant embryos is apparently a consequence of loss of the oNSC pool. (D) A schematic comparing the morphology of the OE, OB, and axonal projections from the OE to the OB via the LOT toward the primary olfactory cortex between control and BAF155cKO mutant embryos. (TIF) [file pgen.1006274.s001.tif]

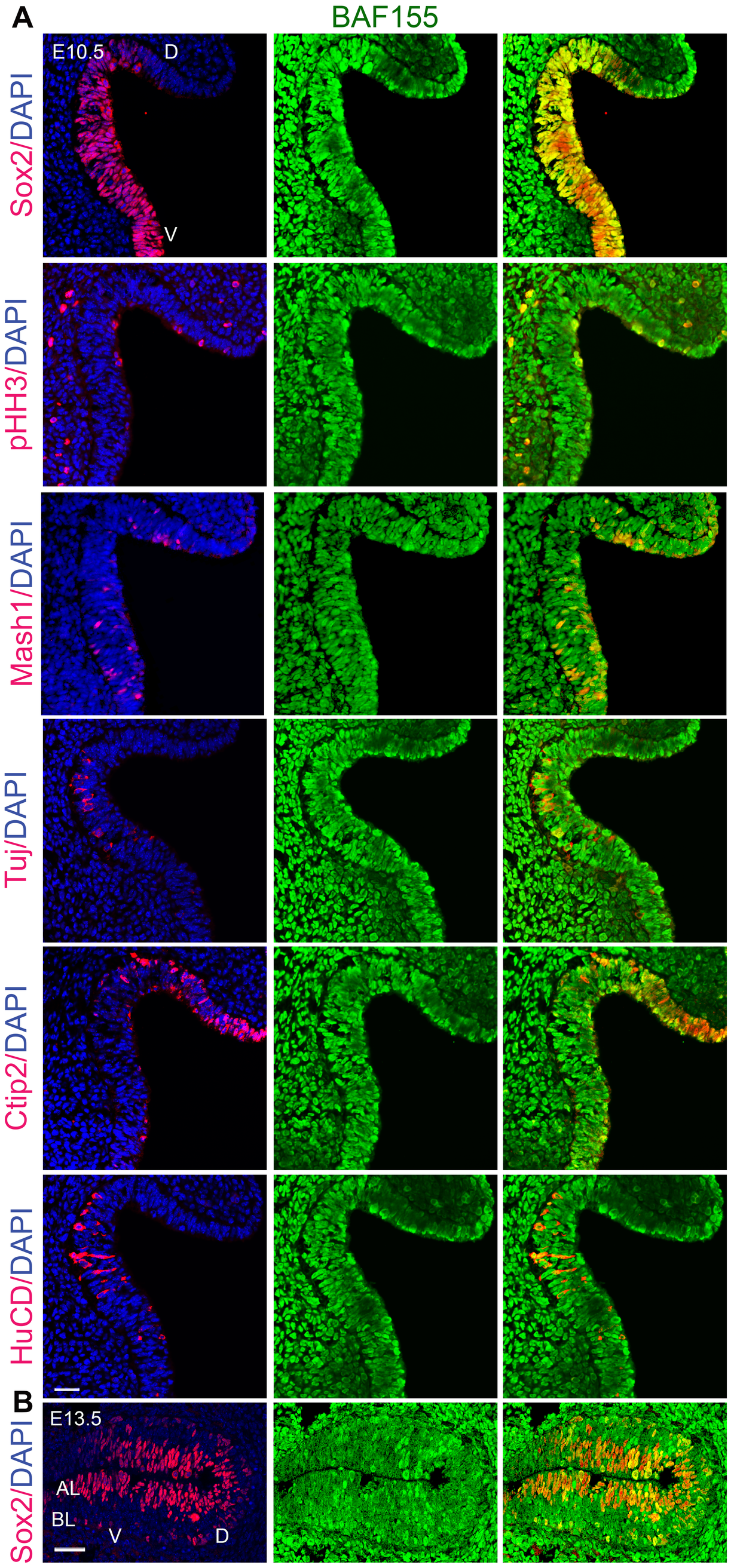

Supplement: S2 Fig — BAF155-expressing cells were characterized in coronal sections of the OE at E10.5 (A) and E13.5 (B) by double-label immunofluorescence microscopy using an antibody for BAF155 (green) in combination with antibodies against the following marker proteins (red): Sox2, in oNSCs at E10.5 (A), and in oNSCs in the basal layer and glia-like SUS cells in apical layers at E13.5 (B); pHH3 in progenitor cells at M-phase of the cell cycle; Mash1 in neuronal progenitors; and Tuj, Ctip2, and HuCD in post-mitotic neurons. Abbreviations: BL, basal layer; ALs, apical layers; D/V, dorsal/ventral. Scale bars = 25 μm (A) and 50 μm (B). (TIF) [file pgen.1006274.s002.tif]

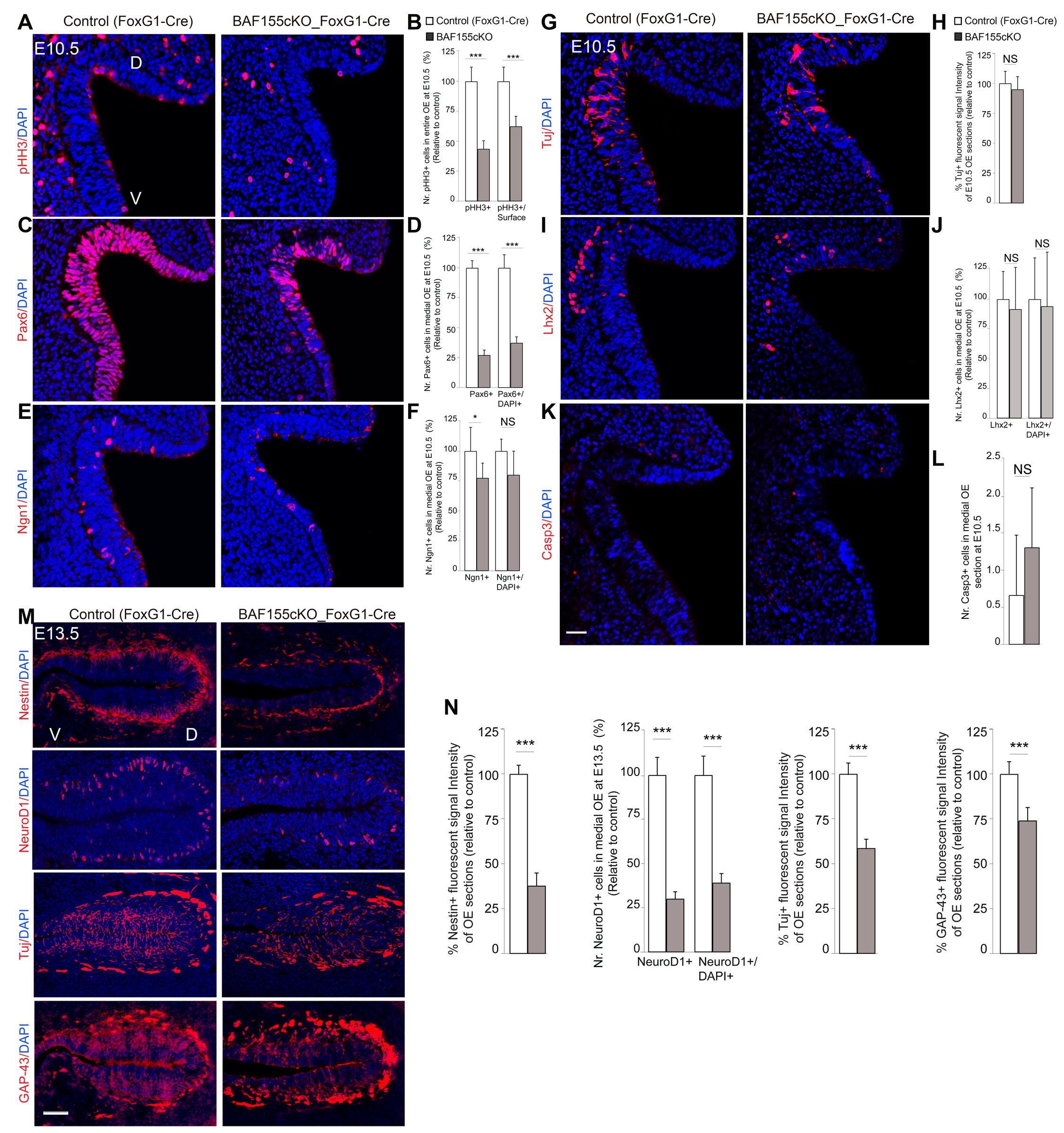

Supplement: S3 Fig — (A–L) IHC (A, C, E, G, I, K) and quantitative (B, D, F, H, J, L) analyses indicate that the loss of BAF155 leads to a diminished number of Pax6+ oNSCs and pHH3+ cells in M-phase of the cell cycle, without affecting Ngn1+ committed neuronal progenitors, Tuj+Lhx2+ neurons, or Casp3+ apoptotic cells at E10.5. Note that quantification of pHH3+ cells (B) was done in the entire OE using 3D reconstruction (see also S1 Movie). (M, N) IHC (M) and quantitative (N) analyses revealed that the expression of Nestin, NeuroD1, Tuj, and GAP-43 at E13.5 is lower in BAF155-deficient OE than in controls. Values are reported as means ± SEM (*P < 0.05, **P < 0.01, ***P < 0.001; NS, not significant). Scale bars = 50 μm (A–K) and 50 μm (M). (TIF) [file pgen.1006274.s003.tif]

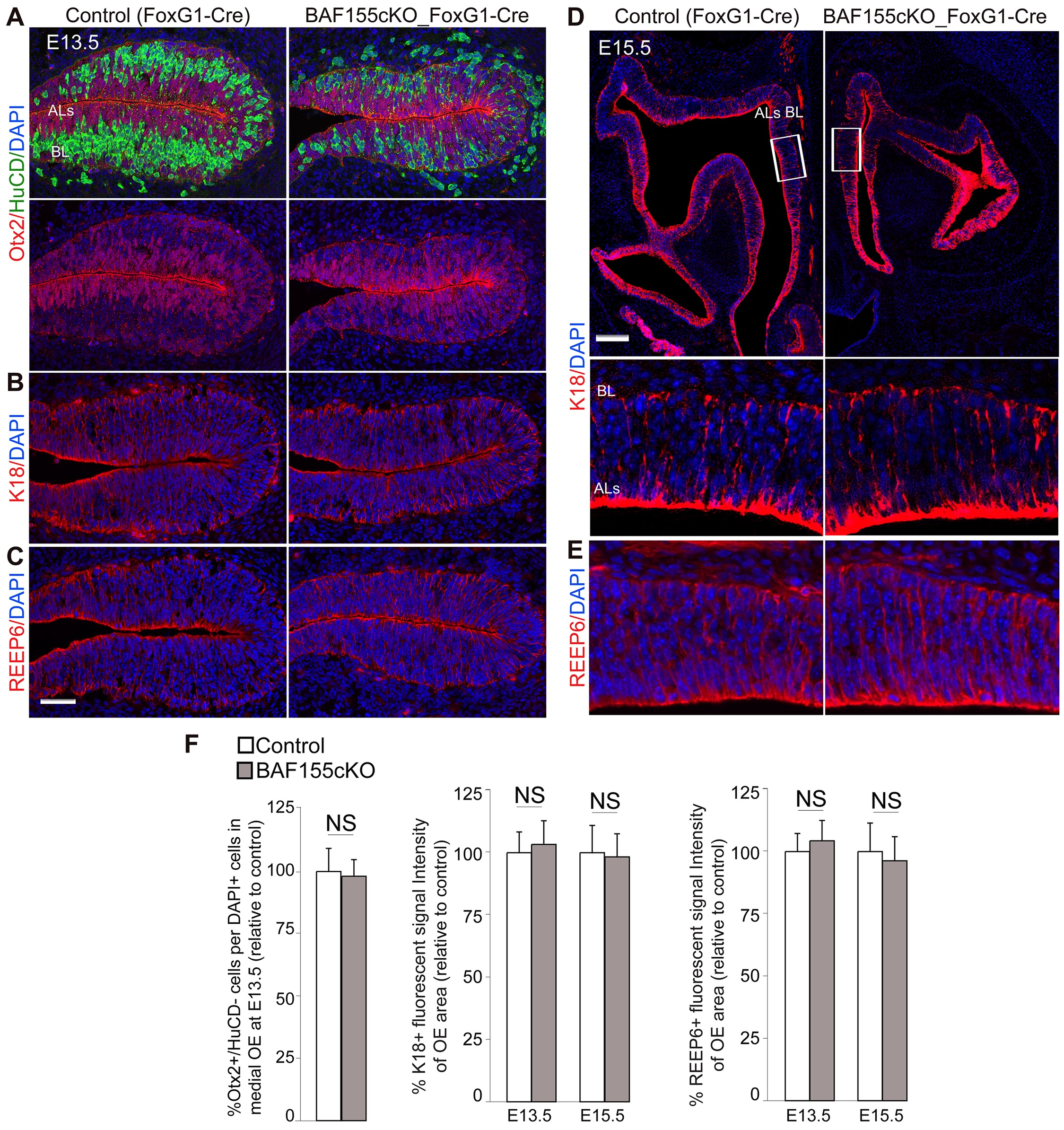

Supplement: S4 Fig — (A–E) Images of IHC with OE section from E13.5 (A-C), E15.5 (D-E) from control and BAF155cKO_FoxG1-Cre embryos and antibodies against: HuCD to labeling ORNs (A), Otx2 to stain both nucleus and cytoplasm of SUS cells [7,37,87] (A), K18 as well as REEP6 to mark SUS cells on the apical layer as well as their basal projections [38]. (F) Quantitative analyses of panels (A-E) indicated that the loss of BAF155 leads to a diminished number of HuCD+ ORNs (A, see also Fig 4), whereas expression of SUS markers: (Otx2, K18, REEP6) were preserved in BAF155-ablated OE as compared to controls. Values are reported as means ± SEM (*P < 0.05, **P < 0.01, ***P < 0.001; NS, not significant). Scale bars = 50 μm (A–C) and 150 μm (D). (TIF) [file pgen.1006274.s004.tif]

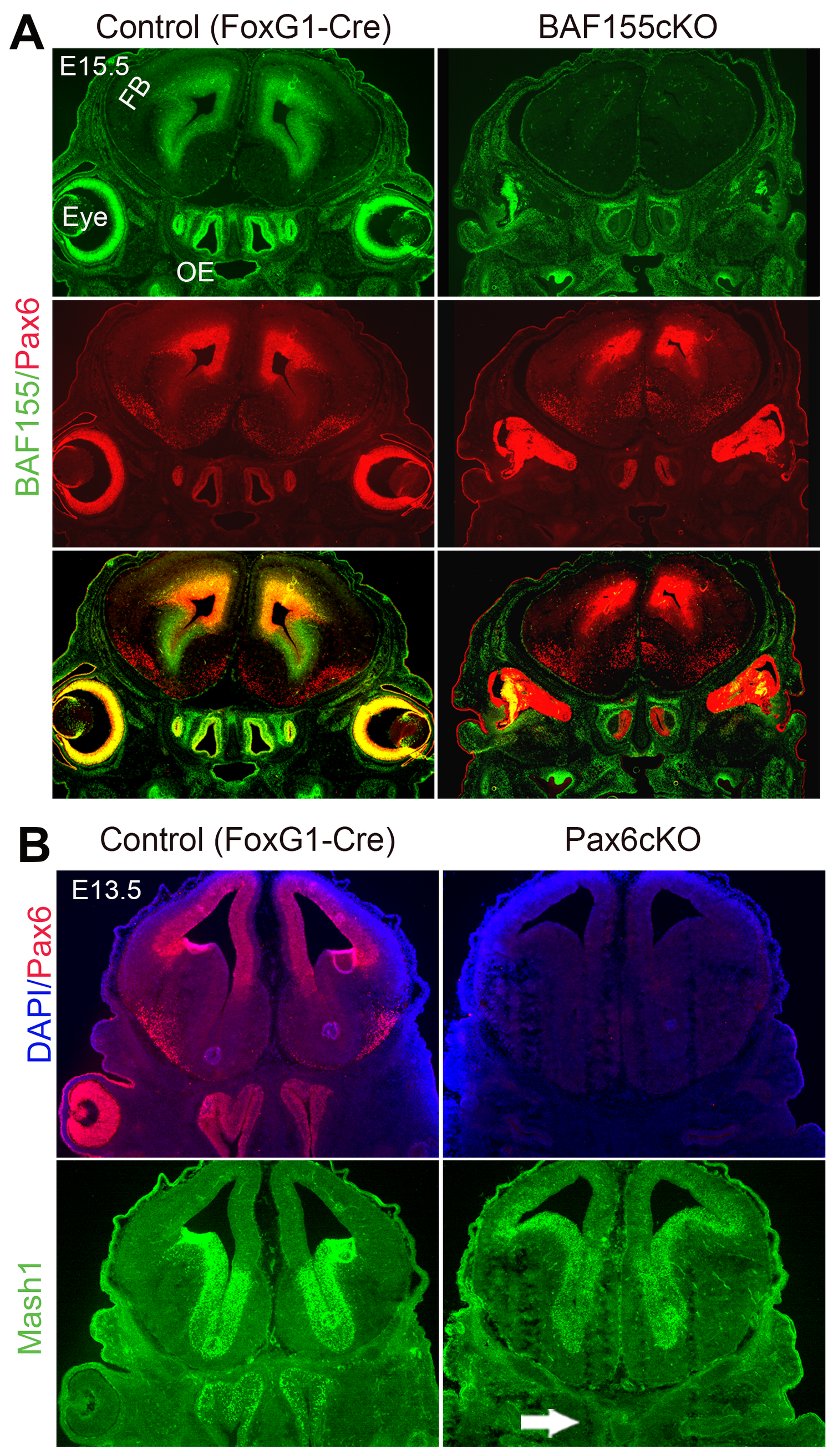

Supplement: S5 Fig — (A) Double-IHC analysis of cross sections of E15.5 heads using anti-BAF155 (green) and anti-Pax6 (red) antibodies indicated a loss of BAF155 expression in the entire forebrain, OE, and eyes of BAF155cKO mice. Although cortex, eye and OE structures are abnormally formed in BAF155cKO mice, the expression of Pax6 is largely preserved. (B) Double-IHC analysis with anti-Pax6 (red) and anti-Mash1 (green) antibodies on cross sections of E13.5 heads revealed a loss of Pax6 expression in the entire head structures of Pax6cKO mice. Similar to Sey/Sey mice, OE (marked by expression of Mash1, arrow) is lost in Pax6cKO mice. (TIF) [file pgen.1006274.s005.tif]

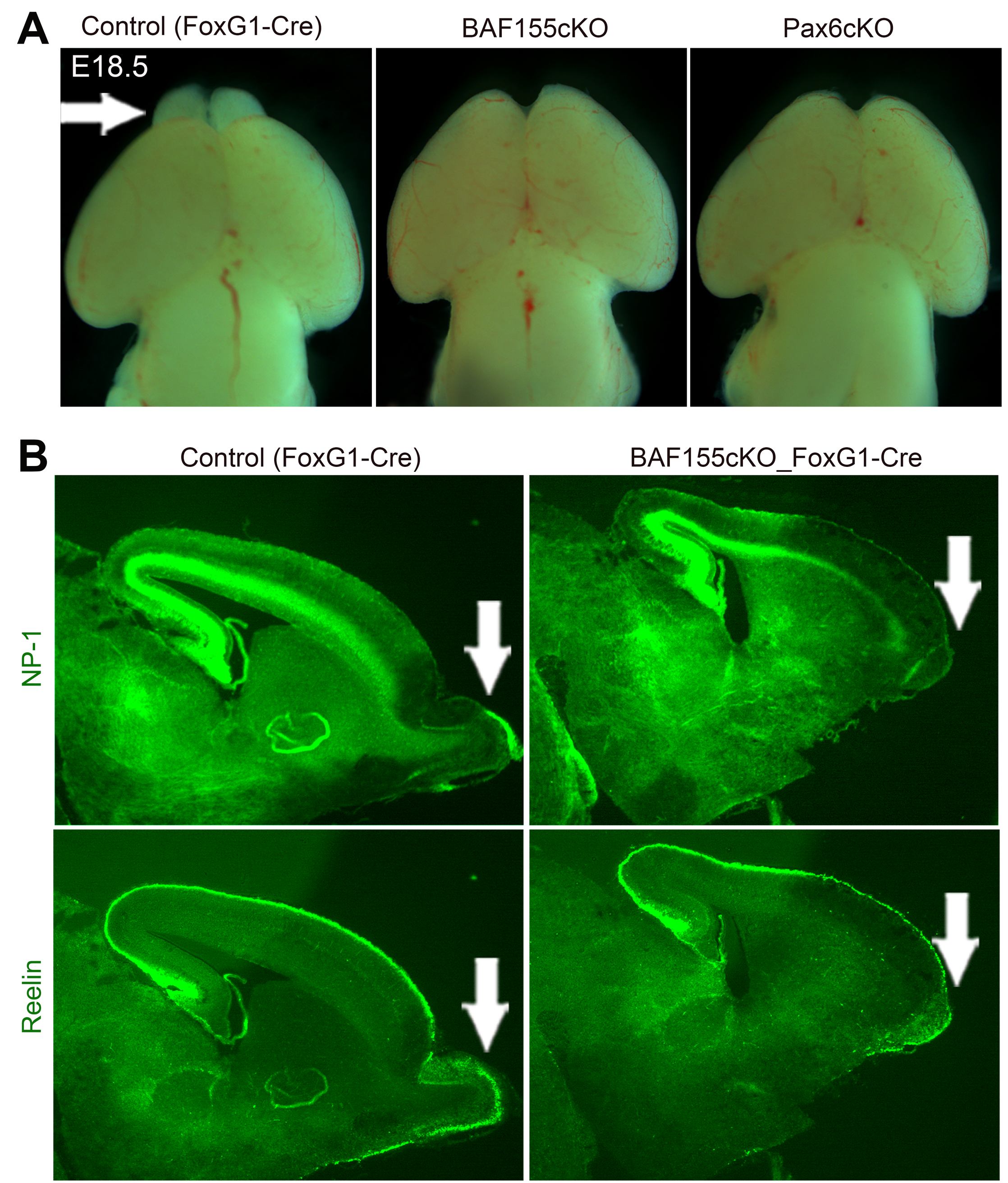

Supplement: S6 Fig — (A) Images of brains (dorsal view) from control and BAF155cKO and Pax6cKO mice at E18.5. Unlike controls, BAF155cKO and Pax6cKO mice lack the OB (indicated by arrow in control). (B) Double-IHC analyses of E18.5 sagittal sections of the brain using anti-NP1 and anti-Reelin antibodies showed that OB formation is induced in the rostral-most telencephalon (arrows) in both control and mutants, but the outgrowth process of the OB is disturbed in BAF155cKO mice. (TIF) [file pgen.1006274.s006.tif]

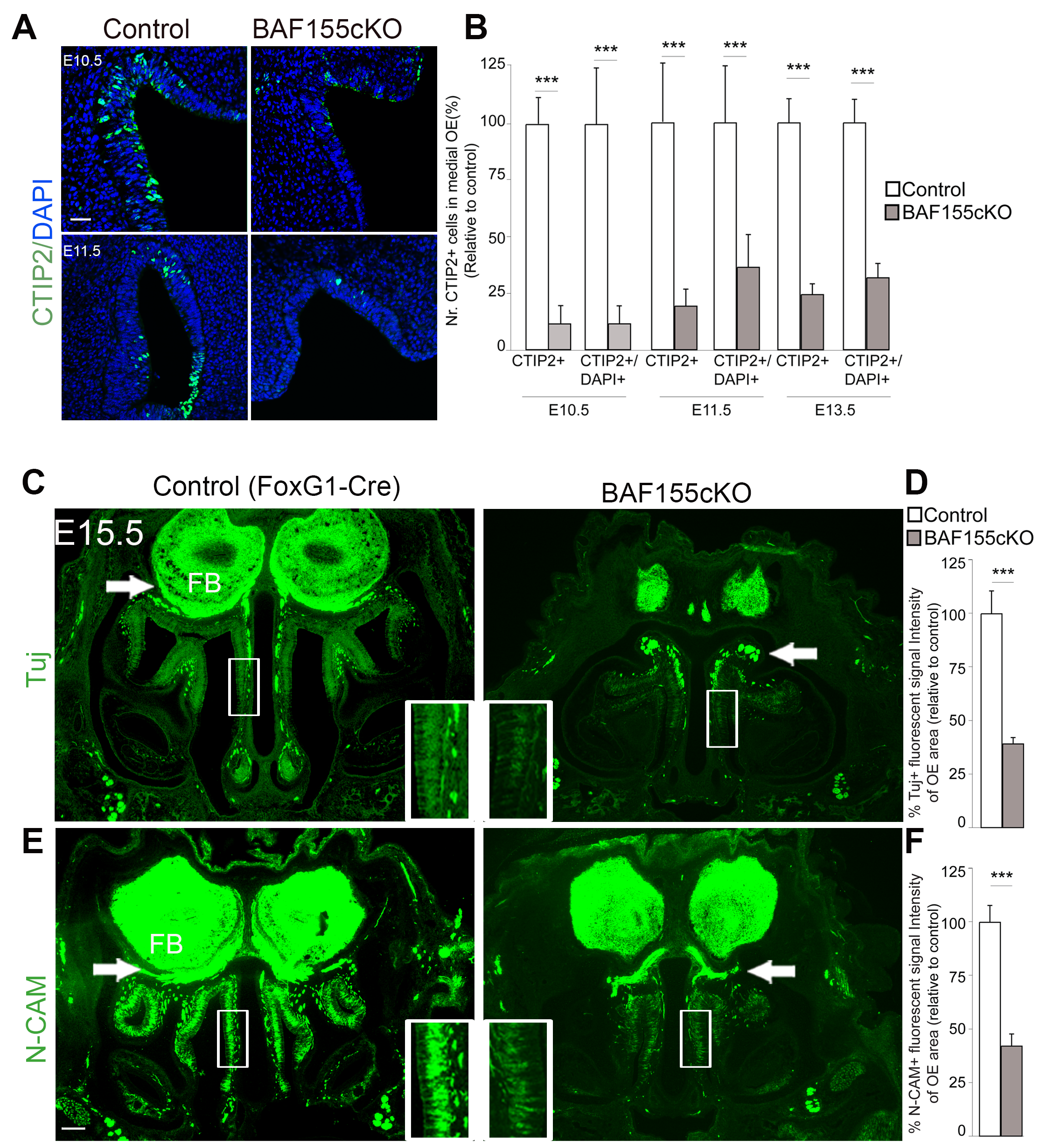

Supplement: S7 Fig — (A, B) IHC (A) and quantitative (B) analyses revealed the loss of Ctip2+ maturing ORNs during OE development (E10.5, E11.5, E13.5). (C–F) IHC (C, E) and quantitative (D, F for selected white boxes in C and E) analyses for the neuronal markers, Tuj (C, D) and N-CAM (E, F) showed reduced expression of the neuronal markers Tuj and N-CAM in the OE of BAF155 mutants at E15.5, and thinner bundles of axons compared with controls (indicated by arrows in C, E). In contrast to the case in controls, ORN axons in mutants do not make their normal contact with the forebrain, as evidenced by the gap between axonal bundles and the forebrain. Values are reported as means ± SEM (***P < 0.001). Scale bars = 50 μm (A) and 150 μm (C–E). (TIF) [file pgen.1006274.s007.tif]

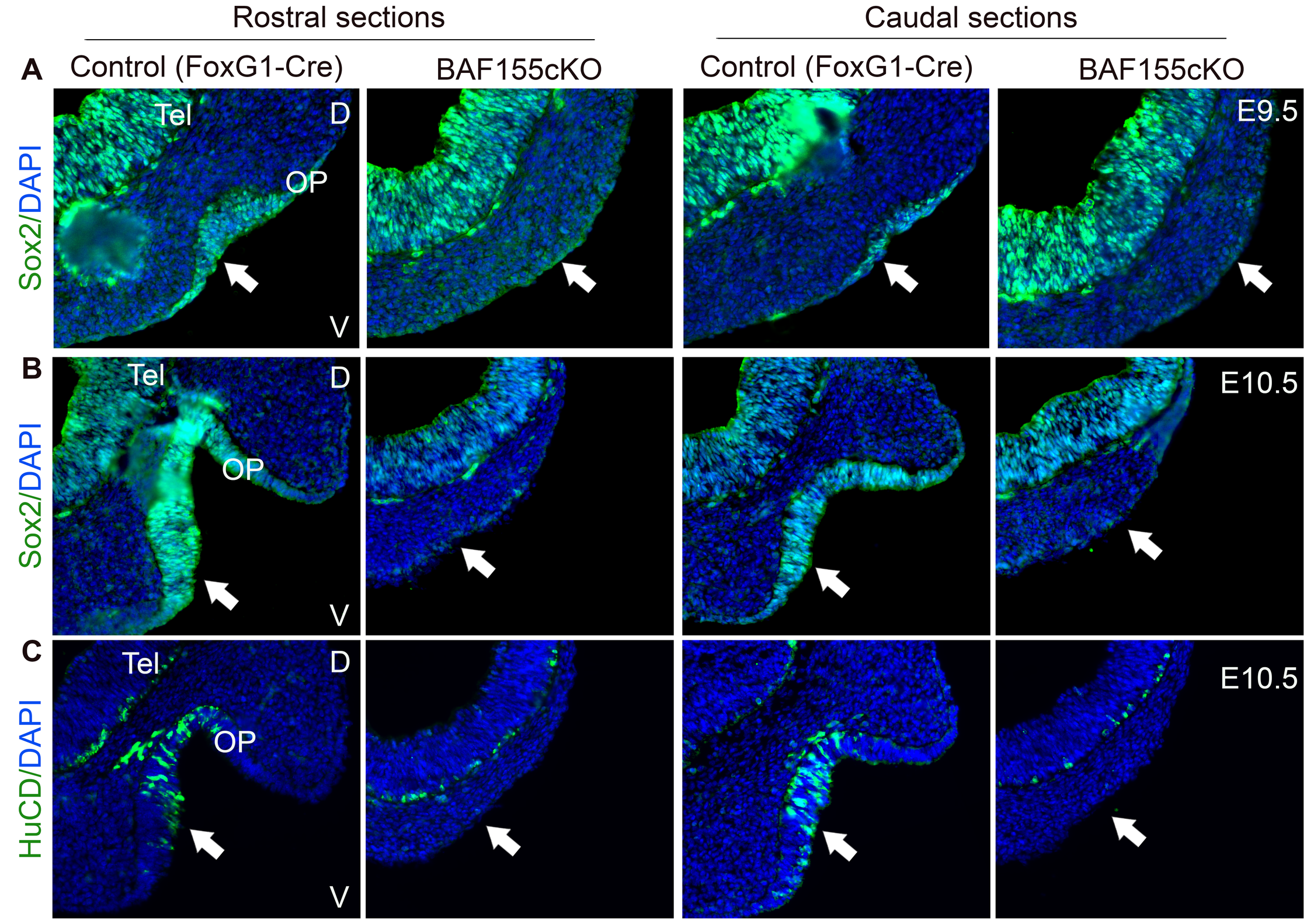

Supplement: S8 Fig — (A-C) Representative images show IHC analyses with coronal sections of olfactory placode (OP) from control and dcKO_FoxG1-Cre embryos at E9.5 (A) and E10.5 (B, C) with antibodies that specifically label primordial oNSC marker Sox2 (A, B) and immature ORN marker HuCD (C). IHC analyses revealed no detectable Sox2+ HuCD+ OP cells (pointed by arrows), implicating that OP/OE was not specified in dcKO embryos. Abbreviations: OP, olfactory placode; OE, olfactory epithelium; Tel, telencephalon; D/V, dorsal/ventral. (TIF) [file pgen.1006274.s008.tif]
