# Supplementary material for: mSWI/SNF (BAF) Complexes Are Indispensable for the Neurogenesis and Development of Embryonic Olfactory Epithelium
Source: PLoS Genet. 2016 Sep 9;12(9):e1006274. doi: 10.1371/journal.pgen.1006274 (PMC5017785; doi:10.1371/journal.pgen.1006274)
Supplement: S1 Text — (DOC) [file pgen.1006274.s011.doc]

#### SUPPLEMENTARY EXPERIMENTAL PROCEDURES

##### Plasmids

Plasmids used in this study (and their origins): pCON/P3 (2xP6CON plus 3xP3 sequences in pGL3 basic, Promega) as described previously .

**Antibodies**

Polyclonal (pAb) and monoclonal (mAb) antibodies used in this study (working dilution; sources): Pax6 mAb (1:200; DSHB), Pax6 rabbit pAb (1:500 for Western blotting and 1:200 for IHC; BABCO), β-actin rabbit pAb (1:1000; Sigma), BAF170 rabbit pAb (Cat. A301-038A, Bethyl), BAF57 rabbit pAb (Bethyl), BAF155 rabbit pAb (Santa Cruz), BAF155 mouse mAb (Santa Cruz), Brg1 rabbit pAb (Santa Cruz), Brg1 mouse mAb (Santa Cruz), Brm mouse mAb (BD Biosciences), Brm rabbit pAb (Abcam), BAF47 rabbit pAb (Abcam), Tuj mAb (1:200; Chemicon), Sox2 mouse mAb (R&D Systems), Ctip2 rat pAb (1:200; Abcam), Mash1 mouse mAb (1:100: BD Biosciences), HuCD mouse mAb (1:50; Invitrogen), phospho-H3 mAb (1:50; Cell Signaling), Ki67 rabbit pAb (1:50; Vector Laboratories), BrdU/IdU mouse mAb (1:40; CalTag), BrdU/CIdU rat pAb (1:100; Abcam), Casp3 rabbit pAb (1:100; Cell Signaling), OMP Goat pAb (Wako), Nestin mouse mAb (BD), GAP-43 rabbit pAb (Santa Cruz), N-CAM mouse mAb (Chemicon), Neuropilin 1 rabbit pAb (Abcam), Casp-3 rabbit pAb (1:100; Cell Signaling), GFP rabbit pAb (1:1000; Abcam), GFP chick pAb (1:1000; Abcam), NeuroD1 goat pAb (Santa Cruz), Otx rabbit pAb (Abcam), K18 rabbit pAb (Abcam), REEP6 rabbit pAb (Proteintech), Luciferase goat pAb (Acris Antibodies), horseradish peroxidase (HRP)-conjugated goat anti-rabbit IgG (1:10000; Covance), HRP-conjugated goat anti-mouse IgG (1:5000; Covance), HRP-conjugated goat anti-rat IgG (1:10000; Covance), and various Alexa-conjugated secondary antibodies (Alexa 488, Alexa 568, Alexa 594, Alexa 647 at 1:400; Molecular Probes). BAF45a, BAF45c, BAF53a, and BAF53b rabbit pAbs were a gift from Dr. Gerald Crabtree (Stanford University), and the Lhx2 rabbit pAb was a gift from Dr. Elaine Fuchs (Rockefeller University).

**Generation of *BAF155*cKO, *BAF170*cKO, *d*cKO mutants**

To eliminate *BAF155* or *BAF170* or both *BAF155* and *BAF170* in the OE, we used *FoxG1*-Cre mice . Heterozygous animals (*BAF155^fl^*^/+^_*FoxG1-*Cre) or (*BAF170^fl^*^/+^_*FoxG1-*Cre) or (*BAF155^fl^*^/+^*,BAF170^fl^*^/+^_*FoxG1-*Cre) were used as controls. BAF155cKO_FoxG1-Cre and dcKO_FoxG1-Cre mutants die soon after birth.

To generate full dcKO_*CAG*-Cre mutants*,* we used the *CAG-CreER* mouse line in which inducible CreER is under the control of the ubiquitous CAG promoter. Homozygous embryos (BAF155^fl/fl^_BAF170^fl/fl^_*FoxG1-*Cre) were used to prepare primary cultured oNSCs.

**IHC and cell-cycle parameters**IHC experiments and determination of cell-cycle index were carried out as previously described .

**Cell counts and quantitative analysis of IHC signal intensity**

IHC quantification was performed using anatomically matched coronal OE sections. Nucleus marker-positive cells within the OE were counted for comparison. In most cases, cell counts of six matched sections in the medial OE were averaged from three biological replicates (control/cKO OE pairs). The number of lineage marker cells was quantified using the total marker-positive cells alone, or by normalizing to the total number of DAPI^+^ (nucleus-stained) cells using the following equation: Normalized number = marker-positive cell number/DAPI^+^ cell number. For quantitative analyses of IHC signal intensity of cytoplasm-stained markers, fluorescent images of entire sections or selected areas of the OE were used. Color images of the OE were converted to gray scale to eliminate background. The fluorescent signal intensity values of pixels were measured using the Analyze/Analyze Particles function of ImageJ software, as previously described , and were expressed relative to normalized values from control experiments as a percentage. Statistical comparisons of histological data were performed using Student’s *t*-test. All bar graphs are plotted as means ± SEM. All statistical tests are two-tailed, and P-values are considered to be significant for α = 0.05. All details of statistical analyses of histological experiments are presented in Table S1.

The relative amount of protein in Western blot experiments was quantified by densitometric analysis of developed films using ImageJ software, as described previously .

**Culture and neuronal differentiation of oNSCs and 4-Hydroxytamoxifen (TAM) treatment**

oNSCs were prepared from the OE of E17.5 and E18.5 WT or *BAF155*cKO_*CAG*-Cre or *d*cKO_*CAG*-Cre embryos as described previously . Briefly, the embryo head was cut through the midline, and the opened midline was positioned facing up, allowing the nasal cavity (rostroventral to the OB) to be identified. The nasal epithelium and surrounding cartilage were excised using microscissors. The OE was dissected away from cartilage under a dissecting microscope. Pieces of the OE were collected in Ca^2+^/Mg^2+^-free HBSS containing 10 mM HEPES. After washing twice with fresh Ca^2+^/Mg^2+^-free HBSS containing 10 mM HEPES, cells were incubated for 10 minutes at 37°C in 0.25% Trypsin/EDTA (Sigma). The tissue was then dissociated mechanically using a serum-coated, fire-polished Pasteur pipette, centrifuged for 5 minutes at 1000 rpm, then washed and resuspended in Dulbecco’s Modified Eagle medium (DMEM) containing 10% fetal bovine serum (FBS). The dissociated cells were cultured on poly-D-lysine–coated plates in serum-free DMEM/Ham's F12 medium, supplemented with insulin, transferrin, selenium, EGF (50 ng/mL), and FGF2 (50 ng/mL). The culture medium was changed every other day. For differentiation of oNSCs into ORNs, cells were cultivated over time in Neurobasal medium containing B-27, penicillin, streptomycin, glutamine, and glutamate.

4-Hydroxytamoxifen (TAM) (H6278, Sigma) was dissolved in ethanol to a final stock concentration of 10 mM (10,000x). Cultured cells were treated with 1 µM TAM or ethanol (vehicle control). During treatment, media were replaced with fresh media containing 1 µM TAM or ethanol every 48 h .

**SUPPLEMENTARY REFERENCES**

1. Tuoc TC, Stoykova A (2008) Trim11 modulates the function of neurogenic transcription factor Pax6 through ubiquitin-proteosome system. Genes Dev 22: 1972-1986.

2. Hebert JM, McConnell SK (2000) Targeting of cre to the Foxg1 (BF-1) locus mediates loxP recombination in the telencephalon and other developing head structures. Dev Biol 222: 296-306.

3. Hayashi S, McMahon AP (2002) Efficient recombination in diverse tissues by a tamoxifen-inducible form of Cre: a tool for temporally regulated gene activation/inactivation in the mouse. Dev Biol 244: 305-318.

4. Tuoc TC, Radyushkin K, Tonchev AB, Pinon MC, Ashery-Padan R, et al. (2009) Selective cortical layering abnormalities and behavioral deficits in cortex-specific Pax6 knock-out mice. The Journal of neuroscience : the official journal of the Society for Neuroscience 29: 8335-8349.

5. Narayanan R, Pirouz M, Kerimoglu C, Pham L, Wagener RJ, et al. (2015) Loss of BAF (mSWI/SNF) Complexes Causes Global Transcriptional and Chromatin State Changes in Forebrain Development. Cell reports 13: 1842-1854.

6. Tuoc TC, Boretius S, Sansom SN, Pitulescu ME, Frahm J, et al. (2013) Chromatin regulation by BAF170 controls cerebral cortical size and thickness. Developmental Cell 25: 256-269.

7. Nguyen H, Sokpor G, Pham L, Rosenbusch J, Stoykova A, et al. (2016) Epigenetic regulation by BAF (mSWI/SNF) chromatin remodeling complexes is indispensable for embryonic development. Cell cycle.

8. Tuoc TC, Stoykova A (2008) Trim11 modulates the function of neurogenic transcription factor Pax6 through ubiquitin-proteosome system. Genes & development 22: 1972-1986.

9. Girard SD, Deveze A, Nivet E, Gepner B, Roman FS, et al. (2011) Isolating nasal olfactory stem cells from rodents or humans. Journal of visualized experiments : JoVE.

10. Stamegna JC, Girard SD, Veron A, Sicard G, Khrestchatisky M, et al. (2014) A unique method for the isolation of nasal olfactory stem cells in living rats. Stem cell research 12: 673-679.

11. Gong Q (2012) Culture of mouse olfactory sensory neurons. Current protocols in neuroscience / editorial board, Jacqueline N Crawley [et al] Chapter 3: Unit3 24.
